# Modelling Robust Feedback Control Mechanisms That Ensure Reliable Coordination of Histone Gene Expression with DNA Replication

**DOI:** 10.1371/journal.pone.0165848

**Published:** 2016-10-31

**Authors:** Andrea Christopher, Heike Hameister, Holly Corrigall, Oliver Ebenhöh, Berndt Müller, Ekkehard Ullner

**Affiliations:** 1 School of Medicine, Medical Sciences and Nutrition, Institute of Medical Sciences, University of Aberdeen Foresterhill, Aberdeen, Scotland, United Kingdom; 2 Department of Physics (SUPA) and Institute for Complex Systems and Mathematical Biology (ICSMB), University of Aberdeen, Aberdeen, Scotland, United Kingdom; 3 Institute of Quantitative and Theoretical Biology, Cluster of Excellence on Plant Sciences (CEPLAS), Heinrich-Heine-Universität Düsseldorf, Düsseldorf, Germany; University College London, UNITED KINGDOM

## Abstract

Histone proteins are key elements in the packing of eukaryotic DNA into chromosomes. A little understood control system ensures that histone gene expression is balanced with DNA replication so that histone proteins are produced in appropriate amounts. Disturbing or disrupting this system affects genome stability and gene expression, and has detrimental consequences for human development and health. It has been proposed that feedback control involving histone proteins contributes to this regulation and there is evidence implicating cell cycle checkpoint molecules activated when DNA synthesis is impaired in this control. We have developed mathematical models that incorporate these control modes in the form of inhibitory feedback of histone gene expression from free histone proteins, and alternatively a direct link that couples histone RNA synthesis to DNA synthesis. Using our experimental evidence and related published data we provide a simplified description of histone protein synthesis during S phase. Both models reproduce the coordination of histone gene expression with DNA replication during S phase and the down-regulation of histone RNA when DNA synthesis is interrupted, but only the model incorporating histone protein feedback control was able to effectively simulate the coordinate expression of a simplified histone gene family. Our combined theoretical and experimental approach supports the hypothesis that the regulation of histone gene expression involves feedback control.

## Introduction

### Histone proteins associate with DNA to form chromatin

Packaging of the genetic material DNA with histone proteins into chromatin is a key feature of eukaryotic cells. Two of the core histone proteins H2A, H2B, H3 and H4 associate with DNA to form the so-called nucleosome core particle, around which 146 base pairs of DNA are wrapped [1]. Such particles are formed at regular intervals and connected by between 10 and 70 base pairs of linker DNA. This packaging is required for higher order chromatin structure formation involving the linker histone H1.

### Histone gene structure and control of expression by modulation of histone RNA levels

In animals, core and linker histone proteins are products of the expression of multi-copy replication-dependent histone genes (referred to as histone genes). DNA replication and histone protein synthesis are synchronised during the cell cycle and are tightly linked during S phase by multiple modes of regulation affecting primarily histone RNA levels (reviewed in [2–4]). Modulation of transcription rates, histone RNA 3’ end formation and stability contribute to the cell cycle control of histone gene expression. Histone RNA levels are between 15- to 50-fold higher during S phase than in G1 or G2 phase [5–7]. This is achieved by a three to five-fold up-regulation of transcription and a six- to eight fold up-regulation of histone RNA 3’ end formation [6,7]. Inhibition of DNA replication leads to a rapid switch-off of histone gene expression by downregulation of transcription and a selective destabilisation of histone mRNA, presumably to avoid detrimental accumulation of excess histone proteins [4–6,8–10].

### Mechanisms and factors controlling histone RNA levels

Upregulation of histone gene transcription at G1/S phase transition depends on the transcriptional co-activator NPAT [11–16]. NPAT is a major component of nuclear structures that assemble at histone gene clusters called histone locus bodies.

Histone RNA 3’ end formation is low in G1 phase and controlled by a checkpoint [17,18]. Histone RNA 3’ ends are produced by RNA cleavage between two conserved elements in the histone RNA 3’ UTR (untranslated region): an RNA hairpin element and the histone downstream element (HDE). This cleavage produces mRNA that ends a few nucleotides after the hairpin structure [19,20]. It depends on the U7snRNP, which binds to the HDE, and the stem-loop binding protein (SLBP), also known as hairpin binding protein HBP, that binds to the histone RNA hairpin element [21–23]. (For simplicity’s sake we refer to this protein here as SLBP). The histone RNA is cleaved by the nuclease CPSF-73, and several other RNA cleavage-polyadenylation factors are also involved in histone RNA 3’ end formation (reviewed in [4]).

Previous studies have identified two cell cycle regulated components of the histone RNA cleavage reaction: SLBP and the heat-labile factor (HLF), a complex that contains symplekin as heat-labile component [24,25]. SLBP is a key factor in the coordination of histone gene expression with DNA replication. RNAi-mediated depletion of SLBP causes inhibition of histone gene expression and cell cycle arrest in S phase [26,27]. SLBP is further also involved in export and translation of histone mRNA [28–30] and the degradation of histone mRNA upon inhibition of DNA replication [31,32]. SLBP levels are cell cycle regulated and increase 10- to 20-fold in late G1 phase, and decrease again upon exit from S phase [24]. The increase is achieved by upregulation of translation while cyclin A/Cdk1-mediated protein phosphorylation at the end of S phase triggers proteasome-mediated degradation of SLBP [24,33–36]. Degradation of SLBP has been linked to protein isomerisation and dissociation of SLBP-RNA complexes by the prolyl isomerase Pin1 [37,38]. The cell cycle control of HLF, which contains CPSF-73, is poorly understood [4].

### Checkpoint control of histone gene expression

During S phase, histone protein synthesis and DNA replication are coupled by checkpoints in an SLBP-dependent manner. Inhibition of DNA replication causes the activation of checkpoint kinases ATR and DNA-PK and the phosphorylation of UPF1, which increases the affinity for SLBP and promotes mRNA degradation by the recruitment of RNA decay factors [32,39,40].

### Degradation of histone proteins

The mechanisms controlling histone protein levels are poorly understood. In yeast, excess histone proteins are rapidly degraded by ubiquitination-dependent proteolysis [41]. In mammals, histone proteins are degraded by a lysosome-mediated process during senescence and by acetylation-mediated proteasomal degradation during spermatogenesis [42,43].

### Histone RNA levels are maintained by autoregulatory mechanisms that compensate for gene loss or gene gain

In animals, the expression of the multicopy histone genes is orchestrated to ensure appropriate overall synthesis of histone proteins. Depletion of 21 out of 44 histone genes in chicken cells was compensated for by the up-regulation of the remaining histone genes [44], and, depletion of highly expressed H3 genes led to compensatory upregulation of the remaining H3 genes, without affecting the expression of other histone gene types [45]. Conversely, inclusion of additional histone H4 gene copies in mouse cells caused a reduction of endogenous histone H4 gene expression [46]. These observations indicate the presence of mechanisms that coordinate the overall expression of histone genes with DNA replication and maintain the expression of histone gene families at appropriate levels.

### Feedback control of histone gene expression

Earlier work led to the proposal of a negative feedback mechanism for the control of histone gene expression, with excess histone proteins controlling histone RNA synthesis [47,48]. Histone H3 protein was found to bind to its own RNA [49] and biochemical studies demonstrated that free histone proteins stimulate the decay of histone mRNA *in vitro* [50,51]. Significantly, inhibition of DNA replication causes an increase of free histone proteins unincorporated into chromatin [52,53]. However, whether the signal for the inhibitory feedback originates from an excess of free histone proteins or alternatively some mechanism senses an excess of newly synthesised, but unpackaged DNA, cannot be directly derived from the available data.

We therefore constructed two simplified mathematical models for the coordination of DNA replication with histone gene expression during the cell cycle, reflecting two possible origins of the feedback signal. In the first model (termed histone feedback loop model) free histone proteins inhibit the synthesis of histone RNA and stimulate histone RNA degradation. In the second model (termed DNA coupled model) unpackaged DNA stimulates histone RNA synthesis and inhibits its degradation.

We parameterised the models using published data and our own experimental work. Both models reflect the changes in histone gene expression during the cell cycle and are capable of simulating scenarios in which DNA replication is interrupted during S phase. The simplest model variants, which do not distinguish between different histone families, are unable to discriminate between the two regulatory mechanisms. We extended our basic models to include an additional histone family. These model variants can be generalised to include all five histone families. These expanded model variants show a clearly different behaviour for the two assumed regulatory mechanisms. Findings support the hypothesis of a regulatory negative feedback loop that originates from the histone proteins. To further challenge our models, we experimentally perturbed the system by introducing additional histone genes.

## Results

### Mathematical modelling of the coordination of DNA replication with histone gene expression

Our mathematical models describe the basic regulation of histone gene expression during S phase. The models focus on the essential features during mid S phase and omit details during the early transition from G1 into S phase, and later during exit from S into G2 phase. We aimed to create models able to describe the regulation of histone gene expression, and the response to perturbations such as the interruption of S phase, in a time-dependent manner. The models use ordinary differential equations (ODEs) capturing rates changing over time for the four core variables histone RNA (*R*), free histone proteins (*H*), new DNA (*D*) representing new histone binding sites and total new nucleosome packed DNA (*T*). Both DNA related variables *D* and *T* are scaled in terms of DNA needed to accommodate histone proteins (98 bp/histone protein of each type). The other variables of the ODE models are scaled in molecules per cell.

**Fig 1:**
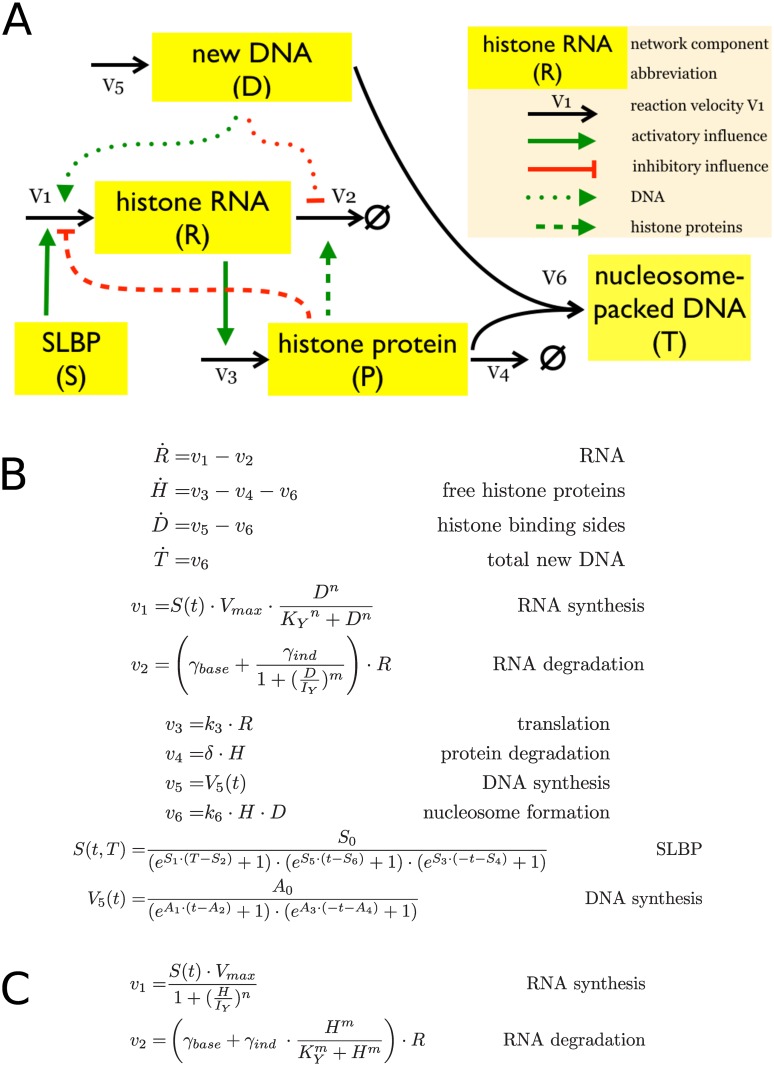
Model structures and equations. Histone feedback loop model and DNA coupled model. (A) Model structure. Dashed lines illustrate the links between free histone proteins and histone RNA synthesis and degradation (histone feedback loop model). The links between DNA replication and histone RNA are illustrated by dotted lines (DNA coupled model). Solid lines are common to both models. (B) and (C). Full set of equations for the histone feedback loop model (B) and the alternative fluxes *v*_*1*_ and *v*_*2*_ for the DNA coupled model (C). Other equations and fluxes are common to both models.

Fig 1 describes two simplified mathematical models. While the components and fluxes are identical, the coordination between DNA replication and histone gene expression is implemented either by i) a negative feedback loop where accumulation of histone proteins inhibits histone gene expression by inhibiting histone RNA production and increasing histone RNA degradation (the histone feedback loop model), as proposed previously [47,48,50,51]; or ii) a direct coupling of DNA replication (*D*) with histone RNA synthesis and degradation (the DNA coupled model), reflecting an S phase checkpoint linking DNA replication with histone gene expression [32,39,40].

The core structure of the two models (Fig 1A) consists of six fluxes (*v*_1_- *v*_6_) that control DNA (*D*) synthesis (*v*_5_), histone RNA (*R*) synthesis and degradation (*v*_1_ and *v*_2_), new histone proteins (*H*) synthesis and degradation (*v*_3_ and *v*_4_), and the capture of histones by the new DNA to form nucleosomes (*T*) (*v*_6_). The stem loop binding protein, SLBP (*S*), is a master regulator of the system and as such is required for the synthesis of histone mRNA. We use the SLBP as an external control parameter defining S phase and disregard the particular regulation of SLBP during early and late S phase. Note the models do not distinguish between the activities of SLBP in histone RNA processing, translation and degradation [21,23,28,29,31,32]. This is justified because all these different activities are ultimately reflected by the dependence of histone RNA synthesis on SLBP, which was observed experimentally [26,27]. The models describe the control of expression of one histone gene family, reflecting the observations that the expression within a gene family is controlled independently of other histone gene types [44–46]. For simplicity’s sake the models are independent of the histone gene numbers. The maximal transcription rate *V*_*max*_ for histone RNA synthesis specifies the overall contribution of all gene copies available. S1 File lists all the parameters used and describes assumptions and necessary approximations.

The four dynamical variables *R*, *H*, *D* and *T* are determined by the six fluxes *v*_1_ –*v*_6_. SLBP *S(t)* and newly synthesised DNA *V*_*5*_*(t)* are time dependent external controls (Fig 1B). The negative feedback loop was implemented by linking histone RNA synthesis (*v*_1_) and degradation (*v*_2_) to free histone protein levels (*H*) (Fig 1A and 1B). Histone RNA synthesis is modulated by the levels of free histone proteins, and histone mRNA degradation is determined by the combination of both basal degradation and induced degradation due to negative feedback from the free histone protein pool. We assume cooperation effects for the RNA synthesis and replication-stress induced RNA degradation and apply Hill functions for these rates, and linear mass-action kinetics for the basal RNA degradation. The regulation of gene expression with feedback loops through Hill functions is a significant simplification but the most widely used approach to model cooperative effects.

**Fig 2:**
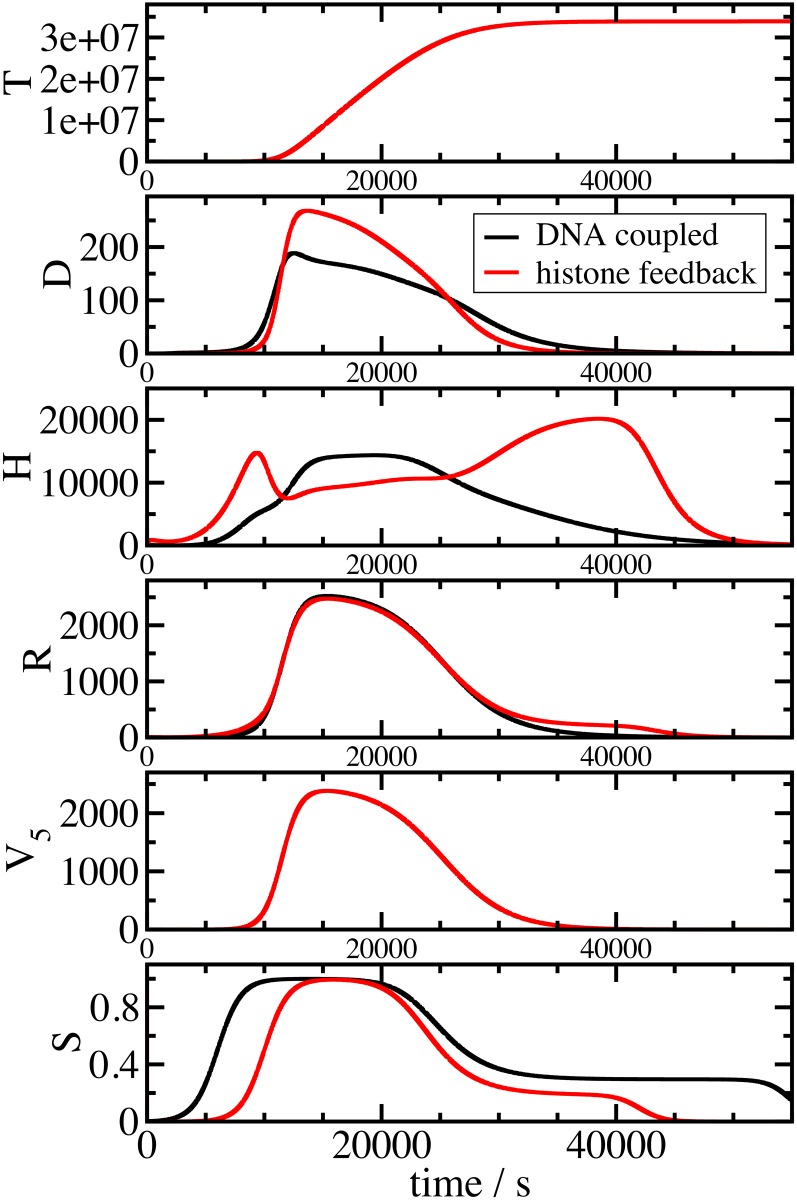
Both models reproduce the cell cycle control of histone gene expression. Panels describe the time evaluation of histone RNA (*R*), histone proteins (*H*), free histone binding sites on DNA (*D*), total new nucleosome packed DNA (*T*) simulated by the mathematical models as a function of the external influence of SLBP (*S*) and DNA synthesis (*V*_*5*_). Shown are results for the histone feedback controlled (red) and DNA coupled (black) model.

In the alternative model, the links between DNA synthesis and histone RNA reflecting a checkpoint that ensures coupling of DNA synthesis with histone gene expression (dotted lines in Fig 1A and fluxes *v*_1_ and *v*_2_ in Fig 1C). The other interactions are as in the histone feedback loop model in Fig 1B. A key external quantity which determines the overall temporal behaviour is the time-dependent DNA synthesis rate *V*_*5*_*(t)*. As external influence on the regulatory mechanism, *V*_*5*_*(t)* is only driven by time and not affected by the histone regulation. This rate was derived from an experimental analysis of DNA content and replication in human immortalised U2OS cells. U2OS cells were synchronised at G1/S phase transition by double thymidine block and then released into S phase. DNA synthesis was monitored by pulse-labelling with BrdU and detected by flow cytometry. *V*_*5*_*(t)* was fitted from the BrdU data using a plateau e-function as described in S2 File and entered into the model (Fig 2, *V*_5_). The plateau e-function produced the best approximation to both data sets available. In parallel, the DNA content was measured in the same cells by staining with *7*-aminoactinomycin D (7-AAD) and analysed and fitted together with the BrdU measurement. This data showed the expected accumulation of DNA during the cell cycle (see Figures E (right) and F (left, red symbols) in S2 File) and matched well the model prediction for new nucleosome packed DNA accumulation (*T*) during DNA replication (Fig 2). A second external quantity is the time-dependent activity of the master regulator SLBP (*S*). This curve was fitted separately for the two models, because its role is different depending on the regulatory mechanism (for details see S1 File).

While both models resulted in similar levels of histone RNA (*R*), which strongly paralleled the experimentally derived *V*_5_, they differed in the production of new histone binding sites (*D*), and the levels of free histone proteins (*H*). In the histone feedback loop model, histone proteins accumulated prior to and after the peak of DNA production at levels above those during peak DNA production, and contributed to the repression of histone synthesis at these stages. In the DNA coupled model, this accumulation was not observed and histone protein levels increased and decreased largely in parallel to DNA synthesis. The difference in timing of the onset and offset of SLBP (*S*) relative to DNA synthesis *V*_*5*_ (Fig 2) reflects the different role of the master regulator in both models and was fitted to allow undisturbed S phase (see S1 File). In mid S phase the shape of the *S* curves coincide in both cases and can therefore not be the origin of any differences between the models at this stage of S phase.

**Fig 3:**
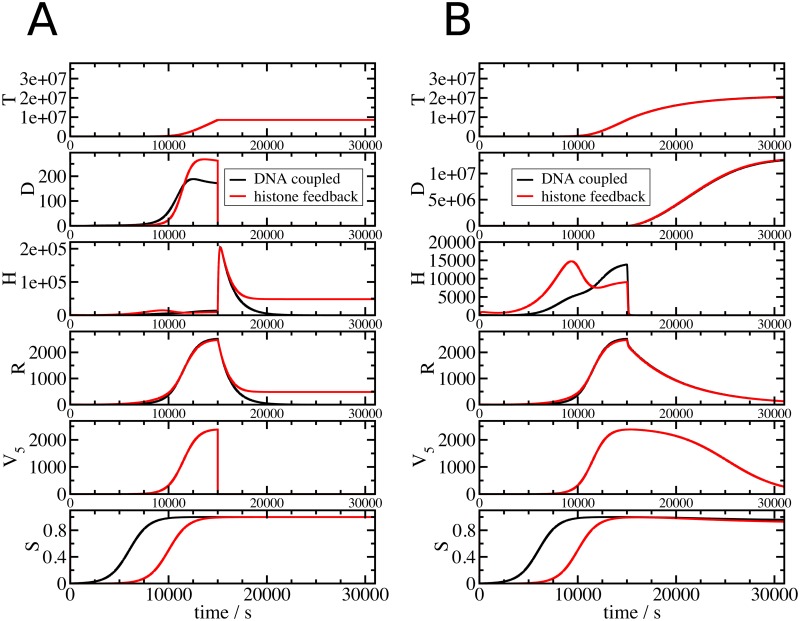
The histone feedback loop and DNA coupled models differ in their response to the inhibition of DNA synthesis but not in their response to transcription blocks. Shown variables and colour coding are as in Fig 2. In (A), inhibition of DNA replication was implemented by setting *V*_*5*_ to 0 at 15000 s. In (B), inhibition of transcription was implemented by setting the transcription flux *v*_*1*_ (see Fig 1) to 0.

Fig 3A displays the results of model simulations, in which DNA was suddenly stopped after *t = 15000s*. It is well established that the inhibition of DNA replication leads to a rapid reduction of histone RNA by a combination of transcription inhibition and histone RNA destabilisation. Clearly, both model versions are able to reproduce this behaviour. Inhibition of DNA synthesis, modelled by reducing *V*_5_ to 0 during S phase, caused in both model variants the termination of DNA synthesis (Fig 3A, *T* and *D*) and a rapid reduction of histone RNA (Fig 3A, *R*) and free histone proteins (Fig 3A, *H*). The reduction of histone RNA results from inhibiting RNA synthesis (*v*_1_) and stimulating RNA degradation (*v*_2_), either by the direct link from new DNA or by the negative feedback from the accumulation of free histone proteins. The flux *v*_2_ is determined by a degradation rate *γ*_*base*_ reflecting the normal histone RNA half-life during S phase, and an enhanced degradation rate *γ*_*ind*_ observed when DNA replication is stopped for example by compounds inhibiting DNA synthesis. We measured both basal and enhanced degradation rates in U2OS cells (S3 File). We used the values *γ*_*base*_ ≈ 0.00018s^-1^ and *γ*_*ind*_ ≈ 0.00067 s^-1^, which correspond to histone RNA half-lives of 64 min and 17.3 min, respectively. These values are in good agreement with previous measurements, reporting histone mRNA half-lives in normal S phase of 45 min in CHO cells [7] and 110 min in HeLa cells [47]. In mouse myeloma and HeLa cells, the histone RNA half-life was reduced to between 10 min and 15 min [8,54] upon interruption of DNA replication.

The model outputs differ however in the levels of histone RNA and proteins after the replication block (Fig 3A, *R* and *H*). While these levels approach 0 in the DNA coupled model they remain clearly higher in the histone feedback loop model. This is necessary for the repression of histone RNA levels by the free-histone-mediated feedback on the RNA synthesis rate *v*_*1*_ and the degradation rate *v*_*2*_. It reflects the standby mode of the histone feedback regulated model with a small pool of histone proteins ready to form chromatin and low level of histone RNA. In both models, the inhibition of DNA replication prevents completion of the duplication of the genome (Fig 3A, *T*), while SLBP levels are not affected by the inhibition of DNA synthesis (Fig 3A, *S*). This is compatible with the observation that the inhibition of DNA replication does not affect SLBP levels [55].

Inhibition of histone gene transcription leads to a different response of the system (Fig 3B). While the free histone pool (*H*) drops down to 0 in both models, DNA replication is not affected and continues. RNA (*R*) decays with a much slower rate than when DNA replication is inhibited as induced RNA degradation is not activated either because of a lack of free histone proteins (histone feedback loop model) or because DNA synthesis continues (DNA coupled model). This means that while the model is able to reproduce the downregulation of histone gene expression when DNA replication is inhibited, it is unable to reproduce the inhibition of DNA synthesis observed when histone gene expression is inhibited (Fig 3B) by for example RNAi knock down of SLBP [26], as the model does not contain a mechanism linking histone gene expression with DNA replication (Fig 1).

**Fig 4:**
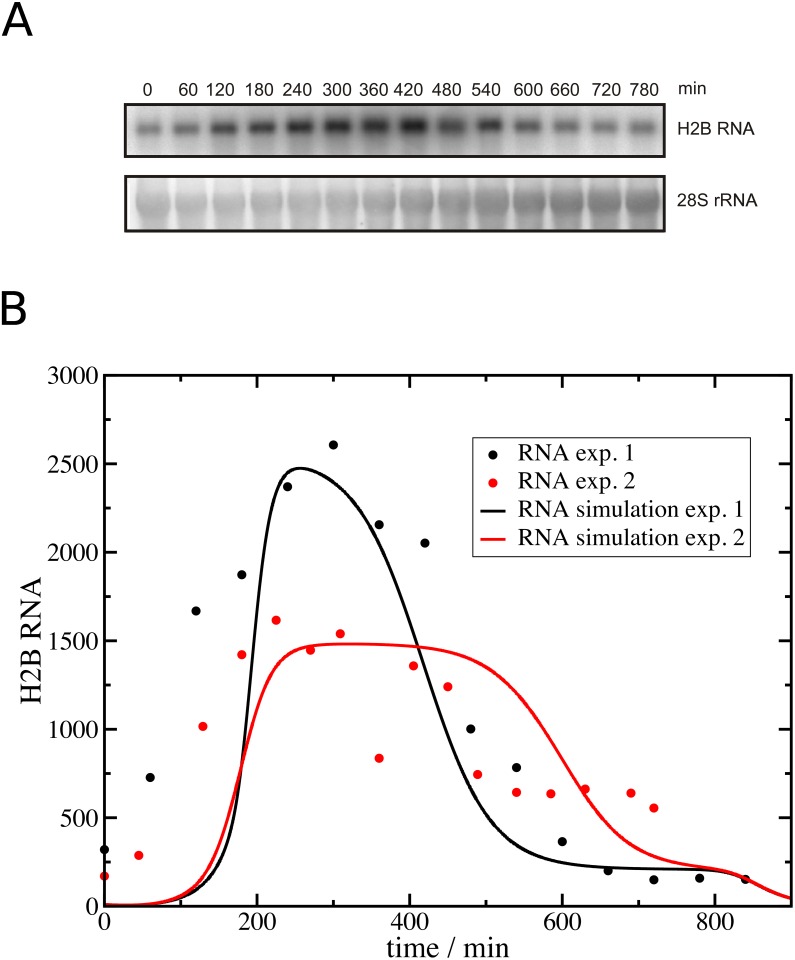
A comparison of histone RNA levels measured experimentally and predicted by the model. (A) U2OS cells were synchronised and released into S phase as described in Materials and Methods. Total RNA was prepared before release into S phase (0 min time point) and at regular intervals after that, and histone H2B RNA levels were subsequently analysed by Northern blotting. 28S rRNA levels were also measured and H2B RNA levels were standardised using 28S rRNA as a reference. (B) The graph shows the model prediction of histone RNA based on the analysis of DNA replication from two independent experiments (RNA simulation exp1 and 2, see S2 File). Also shown is the quantitation of the RNA analysis by Northern blotting from these two experiments (RNA exp 1 and 2). Note these data were scaled to match the maxima of the model predictions.

We then compared histone RNA levels measured experimentally in parallel to DNA replication (Fig 4A) with calculated histone RNA levels based on our cell cycle analysis (S2 File). As shown in Fig 4B the prediction and experimental data correspond reasonably well in the middle of S phase (280 min– 480 min), but they differ significantly in early and late time points. This is also the case in a second experiment for which the prediction of histone RNA levels and measured histone RNA levels are included in Fig 4B.

### Autoregulation of histone gene expression

**Fig 5:**
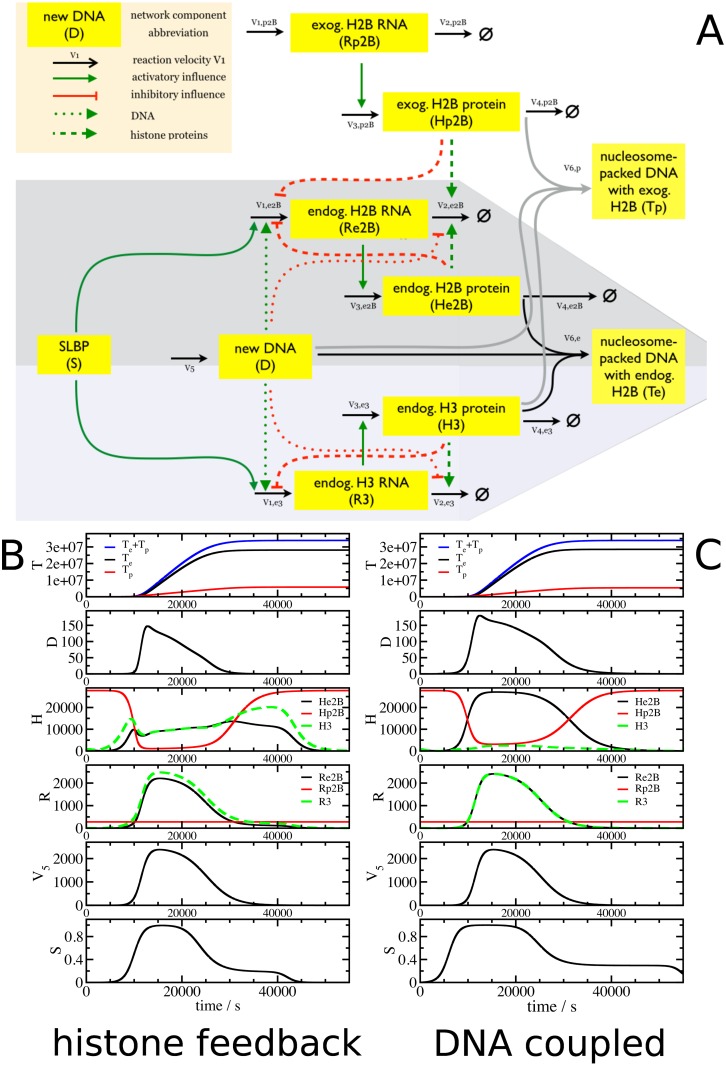
Modelling autoregulation of histone gene expression. (A) Model structure. Expansion of the basic models to test the effect of additional H2B genes on the expression of endogenous H2B and H3 genes. (B) and (C). Panels describe the time evaluation of histone RNA (*R*), histone proteins (*H*), free histone binding sites on DNA (*D*), nucleosome packed DNA formed from endogenous histone H2B and H3 (*T*_*e*_), formed from exogenous H2B and endogenous H3 (*T*_*p*_) simulated by the mathematical models as a function of the external influence of SLBP (*S*) and DNA synthesis (*V*_*5*_). Shown are results for the histone feedback loop (B) and DNA coupled (C) model.

**Fig 6:**
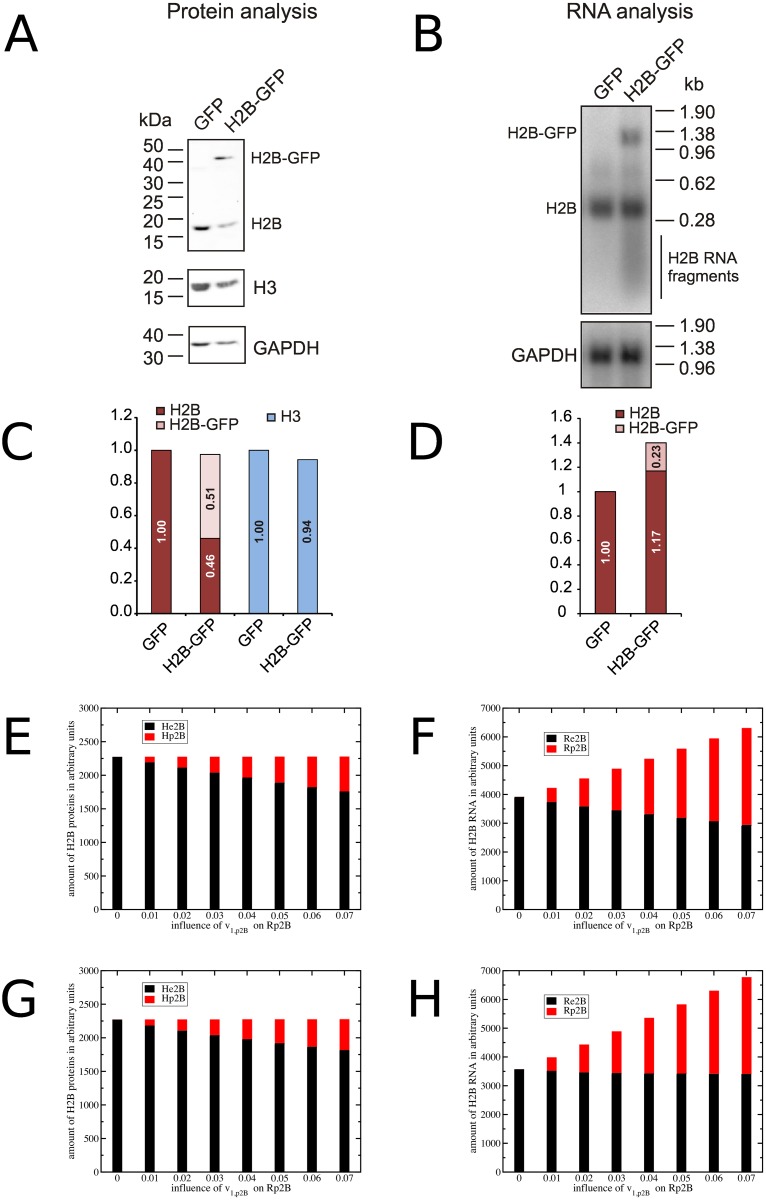
Evidence for a feedback control mechanism that regulates histone gene expression. U2OS cells were transfected with plasmids pEGFP- H2B (H2B-GFP) or pEGFP (GFP) and subjected to antibiotic selection produce stable lines prior to FACS and protein analysis. (A, C) Analysis of H2B protein levels. Proteins were separated by SDS PAGE and analysed by Western blotting. Shown are H2B, H3 and GAPDH protein levels. Histone protein levels were standardised with respect to GAPDH protein, with H2B or H3 protein levels in cells transfected with pEGFP (GFP) defined as 1. Note that endogenous H2B levels (H2B) are significantly reduced in cells expressing H2B-GFP. (B, D) Analysis of H2B RNA levels. H2B RNA levels were analysed by Northern blotting. Shown are H2B and GAPDH RNA levels. The RNA levels were standardised with respect to GAPDH RNA with H2B RNA levels in cells transfected with pEGFP (GFP) defined as 1. Model predictions of the averaged H2B proteins (E, G) and RNAs (F, H) from the histone feedback loop (E, F) and DNA coupled (G, H) model. Endogenous H2B is in black, exogenous H2B in red. The different bars in each plot illustrate the effect of the strength of the promoter controlling exogenous H2B expression.

Histone gene expression is responsive to the loss or gain of histone genes, and compensatory mechanisms ensure that expression is maintained at an appropriate level [44]. Crucially, this occurs within histone gene families without affecting the expression of other histone types [45,46]. Fig 5A illustrates the schematic concept of our experiment and Fig 6A–6D shows experimental evidence for this compensatory mechanism. To introduce additional copies of the histone H2B gene we transfected U2OS cells with a pEGFP derivative plasmid expressing a H2B-GFP fusion protein (pEGFP-H2B) or, as control, with pEGFP only. It is known that GFP-tagged H2B proteins are integrated into nucleosomes [56]. As the H2B-GFP protein is under the control of a viral promoter and polyadenylation signal, it is not subject to histone-specific control. After enrichment of transformed cells by antibiotic selection we confirmed that the selection procedure did not affect cell cycle progression (S1 Fig). The expression of histones H2B and H2B-GFP, and as comparison histone H3, was detected by Western blotting using anti-H2B antibodies (Fig 6A and 6C). H2B protein was detected in extracts from both cell populations while H2B-GFP was detected only in cells transfected with pEGFP-H2B. The quantitation of protein levels showed that histone H3 levels were similar between the two cell populations while the levels of genome-encoded histone H2B was significantly reduced in pEGFP-H2B transfected cells. These cells contained a significant amount of H2B-GFP, which together with the remaining endogenous H2B added up to a similar amount as in the control-transfected cells. This is indicative of an autoregulatory mechanism compensating for loss or gain of histone genes. We also analysed histone H2B RNA levels by Northern blotting (Fig 6B and 6D). Total H2B RNA levels were slightly higher in cells transfected with pEGFP-H2B, mostly due to extra H2B-GFP transcripts. Interestingly we detect some shorter than full-length H2B RNA fragments in cells expressing H2B-GFP, but not in the control cells (Fig 6B). This indicates that increased histone RNA degradation is taking place in these cells, which is compatible with histone mRNA being a target for control of histone gene expression.

Fig 5A describes the structure of the mathematical model expanded to two different histone types, H2B and H3, which together associate with DNA to form nucleosomes. The model allows for alternative and competitive chromatin formation by endogenous or exogenous H2B (*He2B* and *Hp2B*, respectively). It is able to predict the effect of introducing extra histone H2B genes (*Hp2B*) which can associate with histone H3 (*H3)* to form separate nucleosomes (*Tp*).

We compared the ability of histone feedback loop model (Fig 5B) and DNA coupled model (Fig 5C) to meet the experimental results. In both models, H2B-GFP RNA (red curve, *Rp2B*) is constantly expressed and not affected by any feedback control, leading to a similarly constant H2B-GFP RNA level in both models. During S phase, H2B-GFP protein (red curve, *Hp2B*) is incorporated into chromatin (*Tp*) and the level of free H2B-GFP protein is low. Outside S phase, free H2B-GFP protein accumulates and levels are determined by synthesis and degradation. The response of the two models to extra H2B differs. In the histone feedback loop model (Fig 5B), endogenous H2B RNA and proteins are selectively down-regulated (black curves, *Re2B* and *He2B*, respectively) and significantly reduced compared to H3 RNA and protein levels. In contrast, in the DNA coupled model (Fig 5C), an excess of H2B proteins is produced during S phase.

The Northern and Western blots (Fig 6A and 6D) were done with material from non-synchronised cell populations, with cells at all possible stages of the cell cycle. In contrast the models simulate the behaviour of a single cell during S phase (Fig 5B and 5C). Integration of the model predictions over the time of a full cell cycle results in average quantities of a non-synchronised population comparable to the experiments. The strength of the viral H2B-GFP promoter is unknown and so we simulate a range of promoter strengths *V*_*mp2b*_. The bar charts (Fig 6E–6H) show the predicted levels of H2B-GFP and H2B RNA (F,H) and proteins (E,G) for different viral promoter strength *v*_*1*,*p2b*_ derived from the histone feedback (E,F) model and DNA coupled model (G,H). The same amounts of H2B proteins are integrated into chromatin and both models produce a similar outcome. The predictions of RNA levels differ between both control mechanisms. The lack of individual histone type specific regulation in the DNA feedback model results in constant endogenous H2B RNA (*Re2B*) for different plasmid promoter strength (*v*_*1*,*p2b*_). The H2B-GFP RNA (*Rp2B*) adds to the endogenous H2B RNA. The histone feedback mechanism on the other hand leads to a down regulation of the endogenous H2B RNA (*Re2B*) during the S phase. The constant expression of H2B-GFP also beyond S phase leads to an overexpression of the total H2B RNA over the entire cell cycle.

## Discussion

We combined experimental approaches with mathematical modelling to examine and describe quantities which are not experimentally accessible and time evaluations of the regulation of animal histone gene expression. We compared two models where histone synthesis is either controlled by a histone feedback loop or directly coupled to DNA synthesis. We parameterised both models with experimental results and related these back to our experimental data and to reports by others.

Starting with a basic model with only one histone type, both models result in very similar predictions (Figs 1 and 2). Both control mechanisms are able to simulate with reasonable accuracy the levels of histone RNA, the free histone protein pool and the capture of histones by the new DNA to form nucleosomes throughout S phase, and simultaneously avoid excessive accumulation of free histones or a lack of histones, which would slow down DNA replication and thus endanger this vital process. The models are adaptable to different experimental data and the robustness of the models and their parameterisation has been confirmed by parameter variation and sensitivity analysis [57]. Both basic models are a suitable base for further investigations and modifications. The deviation of the model simulations of histone RNA in early S phase from the experimental analysis (Fig 4) might be the result of an intended simplification of RNA synthesis in the mathematical model. We focused the modelling on events occurring in mid S phase rather than on the transition stages when cells enter or exit S phase, and DNA replication changes accordingly. The model assumes that the product of histone RNA synthesis is histone mRNA and does not consider the processing of histone RNA to form functional mRNA, and the export from the nucleus for translation in the cytoplasm. In contrast our analysis of histone RNA by Northern blotting captures precursor RNA, mRNA and RNA destined for degradation located in the cytosol or nucleus. Including these other stages of the histone RNA life cycle into the model may increase its accuracy in early S phase and allow addressing open questions about the role of histone RNA processing, localisation and degradation in the production of histone proteins. This is supported by a time delay in DNA synthesis compared to histone gene expression observed in an analysis of transcriptional and post-transcriptional control of histone gene expression [58].

We tested both models also under extreme perturbed conditions (Fig 3). We blocked in mid S phase either DNA replication or histone RNA synthesis completely, and compared the model results to findings from experiments where these processes were inhibited using either hydroxyurea and/or actinomycin D. Both regulatory mechanisms displayed a very similar behaviour for the inhibition of histone RNA synthesis with actinomycin D. The interruption of DNA replication with hydroxyurea (Fig 3A) led to a downregulation of histone RNA levels and an increased free histone protein pool for the histone feedback loop model whereas the direct DNA coupled model downregulated both histone RNA levels and the histone protein pool. Both regulatory mechanisms prevented the accumulation of superfluous histone proteins, but only in the histone feedback loop model a low amount of histone RNA was maintained. An increase in free histone proteins in cells treated with hydroxyurea has been observed experimentally [52], and hydroxyurea treatment has in many examples caused a severe reduction but not complete absence of histone mRNA [5,40,59]. The presence of low levels of histone RNA and of a pool of histone proteins would be advantageous for a quick resumption of chromatin formation upon release from any DNA replication block. We see this inherent standby feature of the histone feedback loop model as a biologically reasonable advantage over the alternative DNA coupled model.

Artificial overexpression of one histone gene is a strong perturbation of the histone regulatory system to test whether the models are able to simulate the expression coordination of histone gene families [44,45]. Experimentally we introduce additional copies of the histone H2B gene by transfecting U2OS cells with a plasmid expressing a H2B-GFP fusion protein (pEGFP-H2B) or, as control, with pEGFP only. We used the parameterised basic mathematical models as building block to simulate the endogenous histone types H2B and H3 and added a constantly expressed H2B gene to the model for the exogenous H2B gene on the plasmid (Fig 5). The histone feedback loop model predicted a selective downregulation of endogenous H2B RNA and prevented the system from accumulation of free H2B histones. The non-histone type specific regulation of the DNA coupled model was unable to down-regulate the H2B RNA in presence of the exogenous H2B-GFP. Both models differed most in their predictions of the free histone pool during S phase (histone proteins *H* in Fig 5B and 5C). The expression of endogenous histone RNA is another indicator of the different regulatory mechanisms. Experimental time-resolved measurement of the free histone pools of the different types and RNAs during S phase would be a direct test to discriminate between the alternative regulatory mechanisms. However the analysis of the free histone pool is technically challenging.

We compared the different model results with an experimental analysis of histone protein and RNA levels in unsynchronised cells (Fig 6A–6D). Both models indicated similar effects on protein levels. The histone feedback model predicted for non-synchronised cells a partial down regulation of endogenous H2B RNA but not a full compensation of the additional exogenous H2B-GFP RNA because the histone specific regulation is active during S phase and the expression of exogenous H2B-GFP is cell cycle independent. In contrast the DNA coupled model predicted a constant amount of endogenous H2B, with exogenous H2B-GFP expressed in addition. We could clearly detect expression of endogenous H2B RNA and of exogenous H2B-GFP RNA in U2OS cells. As expected, no H2B-GFP RNA was expressed in the control cells. Unfortunately, we were not able to distinguish between these two models as we failed to observe a down regulation of endogenous H2B RNA in cells expressing also H2B-GFP. Interestingly, the Northern Blots reveal an enhanced H2B RNA degradation in the cells expressing H2B-GFP, but not in the control cells expressing GFP (Fig 6B), which is compatible with control of RNA by the histone feedback loop model.

Possibly histone precursor RNAs are targets for enhanced degradation controlled by histone proteins. This, possibly combined with inefficient degradation of these transcripts, perhaps linked to their cellular localisation or to the stage of cells in the cell cycle, may explain the detection of histone RNA degradation intermediate products. But our findings also suggest possible roles for translation control and histone protein turnover in the expression coordination of histone gene families.

The cell cycle and the circadian clock are linked in NIH-3T3 cells, and U2OS cells used in the work described here also have a circadian clock [60,61]. An analysis of RNA levels using CircaDB reveals that some histone RNA levels oscillate with an approximately 24 h periodicity in mouse tissue [62–64]. The RNA levels of the histone gene expression regulators NPAT, SLBP and symplekin lack a clear circadian rhythm; only the RNAs of the U7snRNP components Lsm10 and Lsm 11 oscillate with a 24–28 h periodicity in mouse adipose and lung tissue. These limited observations indicate that histone gene expression may be subject to circadian control, by a mechanism possibly involving the U7 snRNP. Contributions of other factors such as SLBP, which is subject to control at the protein level [24], would need to be investigated to firmly establish a connection between the cell cycle control of histone gene expression and the circadian clock.

In conclusion, we have produced mathematical models for the coordination of histone gene expression with DNA replication during S phase. These models are able to reproduce the key feature of the control of histone gene expression: the link between DNA and histone RNA synthesis. The comparison of model predictions indicates that histone feedback control would be an effective mechanism to coordinate histone gene expression.

## Materials and Methods

### Cell Culture

U20S human osteosarcoma cells were grown in DMEM supplemented with 10% fetal bovine serum (FBS) and streptomycin/penicillin solution (100 mg/ml; 100 U/ml) at 37°C in a 5% CO_2_ atmosphere. U2OS cells were synchronised by double thymidine block. Cells were treated for 17 h with 2 mM thymidine, then released from the block for 10 h, and then treated for 18 h with 2 mM thymidine.

U2OS cells were transfected with pEGFP-N1-H2B plasmids expressing histone H2B tagged with GFP or unmodified pEGFP-N1 (Clontech) using GeneTranIII (Biomiga). After 24 h, cells were grown in DMEM medium supplemented with 10% FBS and 50 μg/ml G418 for 7 days. Effects on cell cycle progression were determined by BrdU labelling and 7-amino-actinomycin D staining followed by FACS analysis. The cells were harvested using trypsin and washed twice with PBS and resuspended in 200 μl. 100 μl of the cell suspension was then mixed with 100 μl SDS-PAGE loading buffer and subjected to Western blot analysis. RNA from the remaining 100 μl was extracted using TRIzol (Life Technologies) and subjected to Northern blot analysis.

### Analysis of DNA replication

DNA replication and cell cycle progression were analysed by flow cytometry using the BD Pharmingen APC BrdU Flow Kit. Cells were labelled for 30 min with BrdU prior and BrdU uptake was subsequently detected using APC conjugated anti-BrdU antibody. 7-amino-actinomycin D (7-AAD) was used to detect the DNA content of the cells. FACS analysis was carried out on a BD Biosciences LSRII flow cytometer at the University of Aberdeen Iain Fraser Cytometry Centre.

### Northern Blot Analysis of Histone RNA Levels

RNA was extracted from U2OS cells using TRIzol according to the manufacturers’ instructions (Life Technologies). Northern blot analysis using 5 μg or 10 μg of total RNA per sample was performed as described previously [26,40]. Histone H2B and GAPDH RNA levels were detected as described using ^32^P-labelled probes [26,40]. 28S rRNA was detected using ^32^P-end-labelled oligonucleotide AACGATCAGAGTAGTGGTATTTCACC [65]. Hybridisation of probes to membranes was carried out according to standard procedures [66]. The RNAs was visualised using a Fujifilm FLA3000 phosphorimager and analysed using AIDA 2.0 software (Raytest GmbH).

### Western Blot Analysis of Histone Protein Levels

U2OS cells transfected with pEGFP-N1-H2B or pEGFP-N1 and control cells were detached, washed in PBS, collected by centrifugation and lysed by re-suspension in 100 μl cell lysis buffer (Cell Signalling Technologies) supplemented with protease inhibitor cocktail (Roche) on ice for 15 min. Protein concentrations were determined by Bradford assay (Bradford 1976), using bovine serum albumin as standard concentrations. Proteins were separated by 15% SDS-PAGE and transferred onto Whatman Protran BA83 Nitrocellulose membrane using Towbin transfer buffer (25 mM Tris, 192 mM glycine and 20% methanol pH 8). Proteins were detected using rabbit anti-H2B antibody (Cell Signalling Technologies), mouse/rabbit anti-H3 (abcam) and mouse anti-GAPDH antibody (Thermo Scientific), and corresponding HRP-coupled secondary antibodies (Cell Signalling Technologies). Protein signals were visualised using a myECL Imager (Thermo Scientific) and proteins bands were quantified using Image Studio Lite software (LI-COR).

## Supporting Information

S1 Fig H2B-GFP expression does not affect the cell cycle of U2OS cells. U2OS cells transfected with plasmids pEGFP- H2B (H2B-GFP) or pEGFP (GFP) were subjected to antibiotic selection to produce stable lines. Asynchronous growing cells were pulse labelled with BrdU for 30 min, fixed, and then stained with 7-AAD to measure DNA replication and content. Staining and analysis by flow cytometry were done as described in Materials and Methods. Cell populations used for the quantitation of the cell cycle analysis are indicated.
(PDF)

S1 File Parameterisation details. (PDF)

S2 File The post processing of the flow cytometry data from synchronised cell cultures to derive the *V*_*5*_ time course for the mathematical model. (PDF)

S3 File The data analysis of the Northern Blots and the derivation of the degradation constants for the mathematical model. (PDF)
