# Supplementary material for: Modelling Robust Feedback Control Mechanisms That Ensure Reliable Coordination of Histone Gene Expression with DNA Replication
Source: PLoS One. 2016 Oct 31;11(10):e0165848. doi: 10.1371/journal.pone.0165848 (PMC5087906; doi:10.1371/journal.pone.0165848)
Supplement: S1 Fig — U2OS cells transfected with plasmids pEGFP- H2B (H2B-GFP) or pEGFP (GFP) were subjected to antibiotic selection to produce stable lines. Asynchronous growing cells were pulse labelled with BrdU for 30 min, fixed, and then stained with 7-AAD to measure DNA replication and content. Staining and analysis by flow cytometry were done as described in Materials and Methods. Cell populations used for the quantitation of the cell cycle analysis are indicated. (PDF) [file pone.0165848.s001.pdf]

S1 Fig. H2B-GFP Expression does not affect the cell cycle of U2OS cells.

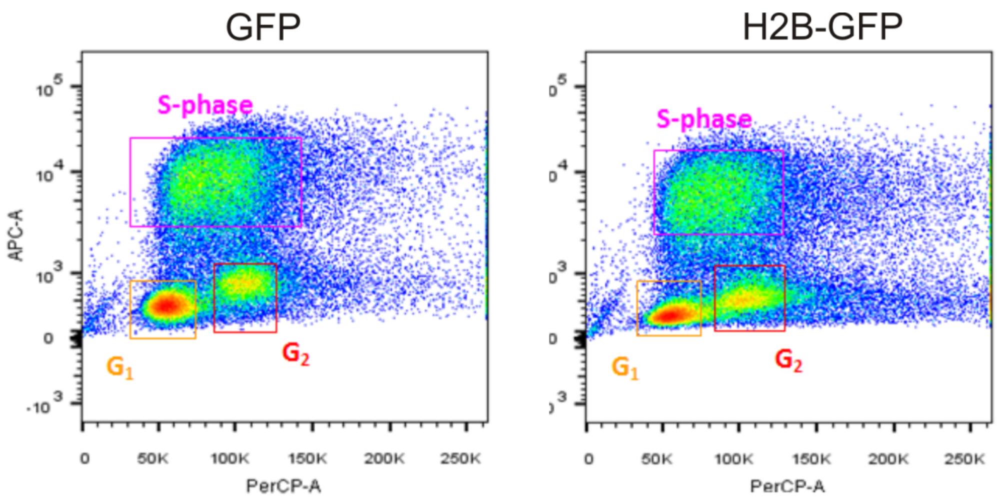

	G1	S	G2
H2B-GFP	23.7%	29.3%	19.0%
GFP	24.1%	31.9%	17.5%

Cell cycle analysis. U2OS cells transfected with plasmids pEGFP- H2B (H2B-GFP) or pEGFP (GFP) were subjected to antibiotic selection to produce stable lines. Asynchronous growing cells were pulse labelled with BrdU for 30 min, fixed, and then stained with 7-AAD to measure DNA replication and content. Staining and analysis by flow cytometry were done as described in Materials and Methods. Cell populations used for the quantitation of the cell cycle analysis are indicated.
