# Supplementary material for: Modelling Robust Feedback Control Mechanisms That Ensure Reliable Coordination of Histone Gene Expression with DNA Replication
Source: PLoS One. 2016 Oct 31;11(10):e0165848. doi: 10.1371/journal.pone.0165848 (PMC5087906; doi:10.1371/journal.pone.0165848)
Supplement: S1 File — (PDF) [file pone.0165848.s002.pdf]

S1 File: Parameterisation details

Parameter	regulation		source
	histone feedback	DNA coupled	
V_{max}	$15\ s^{-1}$		calculated
K_Y	1240		estimated
I_Y	1240		estimated
n	1		estimated
m	1		estimated
γ_{base}	$0.00018\ s^{-1}$		experiment, see supplementary S3 File
γ_{ind}	$0.00067\ s^{-1}$		experiment, see supplementary S3 File
k_3	$1\ s^{-1}$		calculated, [1]
δ	$0.01\ s^{-1}$		estimated
k_6	$0.001\ s^{-1}$		chromatin synthesis [2]
A_0	$2470\ s^{-1}$ / $1330\ s^{-1}$		experiment, derived from the BrdU flow cytometry data from experiment 1 (left) and experiment 2 (right), for details see the supplementary S2 File
A_1	$0.000361\ s^{-1}$ / $0.00037\ s^{-1}$		
A_2	$25200\ s$ / $36400\ s$		
A_3	$0.00126\ s^{-1}$ / $0.000833\ s^{-1}$		
A_4	$-11500\ s$ / $-10800\ s$		
S_0	$1\ s^{-1}$		arbitrarily chosen to construct a meaningful master regulation by SLBP, for details see the description below
S_1	0.0000003		
S_2	29000000	31000000	
S_3	$0.001\ s^{-1}$		
S_4	$-10000\ s$	$-6000\ s$	
S_5	$0.001\ s^{-1}$		
S_6	$42000\ s$	$55000\ s$	

Table A: Parameter of the two basic feedback models.

Biological process	Amount	Literature source
Transcription	*70 nt/s*	[2]
Translation	*60 nt/s; 5 s/mRNA*	[1]
Replication	*100 nt/s (eukaryotes)*	[4]; [5]
Chromatin synthesis	*1100 s^{-1} (0.001 s)*	[2]
Open reading frame	*300 nt*	[2]
Footprint	*~50 nt*	[1]
mRNA half-life period	*10-45 min*	[6,7] [8]
Haploid human genome size	*3.4×10^9*	[5]
Amount of each core histone	*3.4×10^7*	[2]
S phase	*8 h ~ 30000 s*	[4]

Table B: Values for parameterisation of the model also described in [3] .

Calculation of maximal transcription rate V_{max}

$$Transcription\,rate = \frac{Transcription}{Footprint} = \frac{70nt/s}{50nt} \approx 1s^{-1}$$

The transcription rate of a singe copy is scaled by the number of gene copies, e.g. 15 genes of the H4 and H2B genes. The other core histone genes are similar frequently present on the genome (12 H3 and H2A).

$$V_{max} \approx 1s^{-1} \times 15 \approx 15\,s^{-1}$$

Calculation of the translation rate k_3

$$Translation\,rate\,k_3 = \frac{Translation}{Footprint} \approx \frac{\frac{60nt}{s}}{50nt} \approx 1s^{-1}$$

Calculation of the histone binding site replication rate k_5

Because of our detailed measurement of the time-dependent DNA production rate V_5, we do not actually use this value in the model. However, an estimate of the average production rate of histone binding sites is useful for comparison with our measured rate V_5 and to justify the parameter value for A_0. If the DNA replication takes place constantly over the entire S phase the average rate of incorporation of histone proteins into chromatin can be estimated as

$$Replication\ rate\ k_5 = \frac{Amount\ of\ histones}{length\ of\ S\ phase} \approx \frac{3.4 \times 10^7}{30000s} \approx 1200s^{-1}$$

The master SLBP regulation *S(t,T)*

The master regulation determines the S phase in the mathematical model and is an external time dependent control parameter for our mathematical model. The SLBP regulation is not the focus of our studies and affects the dynamics of the model only early and late in S phase. It has more importance for the histone feedback loop model than for the DNA coupled model. We model the time course of SLBP with plateau e-functions similar to the equation of the DNA replication $V_5(t)$ and apply it as a necessary factor for histone RNA synthesis. We choose the parameters S_1-S_6 arbitrarily so that SLBP is not limiting during full S phase. The histone feedback loop model is in standby in the absence of DNA synthesis, and SLBP controls beginning and end of S phase. The DNA coupled model has no standby mechanism. The different regulatory mechanisms imply individual choices of the parameters controlling SLBP. We required for the S_1-S_6 parameter selection that the DNA checkpoint and histone feedback regulated models are able to produce meaningful results for both experimental $V_5(t)$ curves derived from the BrdU flow cytometry data from experiment 1, described in details in the supplementary S2 File, and experiment 2 with 45 min sampling. Further we required from the particular S_1-S_6 parameter selection that both models behave similar in the measurable quantities of histone RNA (R) and total new nucleosome packed DNA (*T*). The plateau e-function is built by three factors in the denominator, the time (*t*) controlled rising term $(e^{S_3(-t-S_4)} + 1)$, the time controlled decay phase to the end of S phase $(e^{S_5(t-S_6)} + 1)$ and the SLPB down regulated by accomplishing the DNA replication $(e^{S_1(T-S_2)} + 1)$. SLPB is either downregulated because the maximal time length of S phase has been reached or when DNA replication and nucleosome packaging (*T*) is complete. Examples for the differently chosen SLBP curves in the histone feedback loop model and the DNA coupled model are shown in Figure 2. We chose SLBP to rise later in the histone feedback loop model than in the DNA coupled model for the reasons outlined below. In the DNA coupled model we have two positive influences, from DNA synthesis and SLBP, on histone RNA synthesis, and both are required. To avoid a lack of histone proteins in early S phase in this model, SLBP must increase before DNA replication, to allow the model to respond sensitively and quickly to the demand created by DNA synthesis. The histone feedback loop model controls the histone RNA synthesis by two antagonistic influences, the positive effect of the SLBP master regulation and the inhibition by the free histone protein pool. This regulation leads to the standby mechanism where free histone proteins are present, as soon SLBP is produced at the beginning of

S phase. Simulating the SLBP onset as early as in the DNA coupled model would advance and extend the standby phase in early S phase but would not have an effect on the chromatin formation. As mentioned before the early S phase and the detailed effect of SLBP are not at the centre of our investigations. The different choices for S_1-S_6 do not affect the regulation in mid S phase. Our experiments and model calculations focus on mid S phase and are not limited by the particular chosen on- and offset of the SLBP curve *S(t,T)* in both mathematical models.

General remarks on the basic model

Our numerical studies are designed to compare conceptually different regulatory mechanisms. In particular, they do not follow the aim to examine small quantitative differences resulting from particular parameter choices. We apply similar simplifications to both models. Especially the approximation of the various regulated steps of transcription and translation by Hill functions is a significant simplification but justified to investigate the main question regarding regulatory principles. For parameterisation, we use our own experimental measurements or literature values if available (see table above). The parameters defining the Hill type RNA synthesis and induced degradation (K_Y l_Y n and m) are ad hoc choices to reach meaningful RNA levels during S phase. A detailed sensitivity analysis and systematic parameter variation has been performed by H. Hameister [3]. A wide range of the parameters allows a reliable and fast regulation of the histone RNA and so the particular choice is not critical for the comparison of the feedback concepts although the non-linear Hill functions and parameters therein have a significant effect on the particular concentrations. Importantly for the present studies, most of the parameters were the same in the two models.

The model could be made more detailed by including the processing of histone pre-mRNA and transport processes from the nucleus and taking into account time delays during the formation of mRNA. These refinements would require more quantitative experimental data to deliver a real additional predictive value compared to our simplified mathematical models.

Extended models of two core histone types H2B and H3 and exogenous GFP-H2B

Both basic models (the DNA coupled model and histone feedback loop model – see Figure 1) can be used as building blocks to construct an enlarged model for the formation of chromatin by combining modules for each of the different histones. The model described here takes into account H2B and H3 proteins and DNA for the formation of chromatin and assumes an identical behaviour for the other

histone proteins H2A and H4. We use the parameters from the basic models (see table above) for the endogenous H2B and H3, i.e. both core histones follow the same dynamics. Only the chromatin synthesis k_6 must take into account the complex formation of three reactants and is set to k_6=0.0000002 s^{-1} to ensure a similar curve of *D* and *T* as in the basic models. The exogenous H2B-GFP RNA is transcribed continuously with a transcription flux V_{1p2B} , degraded with the same basal degradation rate γ_{base} as the endogenous H2B RNA and is isolated from control by histone protein or link to DNA synthesis. Similar to H2B, H2B-GFP is incorporated into chromatin together with H3 and DNA. As a result, one part of the chromatin is GFP labelled and contains H2B-GFP (T_p) and the other part contains endogenous H2B (T_e). We compare the direct DNA coupled model with the histone feedback loop model. The exact H2B-GFP RNA synthesis rate in the experiment is unknown and we test the model with a set of different rates between 0 s^{-1} and 0.07 s^{-1}.

The full set of equations is shown in Figure A for the histone feedback loop model. The alternative DNA coupled model requires only different equations for the fluxes of the endogenous RNA synthesis and degradation. These are shown in Figure B.

$$\dot{R}_{e2b} = v_{1e2b} - v_{2e2b} \quad \text{endog. H2B RNA}$$
$$\dot{R}_{p2b} = v_{1p2b} - v_{2p2b} \quad \text{exog. H2B RNA}$$
$$\dot{R}_{e3} = v_{1e3} - v_{2e3} \quad \text{endog. H3 RNA}$$

$$\dot{H}_{e2b} = v_{3e2b} - v_{4e2b} - v_{6e} \quad \text{free endog. H2B proteins}$$
$$\dot{H}_{p2b} = v_{3p2b} - v_{4p2b} - v_{6p} \quad \text{free exog. H2B proteins}$$
$$\dot{H}_{e3} = v_{3e3} - v_{4e3} - v_{6e} - v_{6p} \quad \text{free endog. H3 proteins}$$

$$\dot{D} = v_5 - v_{6e} - v_{6p} \quad \text{free new DNA binding sides}$$
$$\dot{T}_e = v_{6e} \quad \text{total new DNA built with endog. H2B}$$
$$\dot{T}_p = v_{6p} \quad \text{total new DNA built with exog. H2B}$$

$$v_{1e2b} = S(t) \cdot V_{max,e2b} \cdot \frac{1}{1 + \left(\frac{H_{e2b}+H_{p2b}}{I_Y}\right)^n} \quad \text{endog. H2B RNA synthesis}$$
$$v_{1p2b} = V_{max,p2b} \quad \text{exog. H2B RNA synthesis}$$
$$v_{1e3} = S(t) \cdot V_{max,e3} \cdot \frac{1}{1 + \left(\frac{H_{e3}}{I_Y}\right)^n} \quad \text{endog. H3 RNA synthesis}$$

$$v_{2e2b} = \left(\gamma_{base} + \gamma_{ind} \cdot \frac{(H_{e2b} + H_{p2b})^m}{K_Y^m + (H_{e2b} + H_{p2b})^m}\right) \cdot R_{e2b} \quad \text{endog. H2B RNA degradation}$$
$$v_{2p2b} = \gamma_{base} \cdot R_{p2b} \quad \text{exog. H2B RNA degradation}$$
$$v_{2e3} = \left(\gamma_{base} + \gamma_{ind} \cdot \frac{H_{e3}^m}{K_Y^m + H_{e3}^m}\right) \cdot R_{e3} \quad \text{endog. H3 RNA degradation}$$

$$v_{3e2b} = k_3 \cdot R_{e2b} \quad \text{endog. H2B translation}$$
$$v_{3p2b} = k_3 \cdot R_{p2b} \quad \text{exog. H2B translation}$$
$$v_{3e3} = k_3 \cdot R_{e3} \quad \text{endog. H3 translation}$$
$$v_{4e2b} = \delta \cdot H_{e2b} \quad \text{endog. H2B protein degradation}$$
$$v_{4p2b} = \delta \cdot H_{p2b} \quad \text{exog. H2B protein degradation}$$
$$v_{4e3} = \delta \cdot H_{e3} \quad \text{endog. H3 protein degradation}$$
$$v_5 = V_5(t) \quad \text{DNA synthesis}$$
$$v_{6e} = k_6 \cdot H_{e2b} \cdot H_{e3} \cdot D \quad \text{DNA-histone complex synth. with endog. H2B}$$
$$v_{6p} = k_6 \cdot H_{p2b} \cdot H_{e3} \cdot D \quad \text{DNA-histone complex synth. with exog. H2B}$$

$$S(t) = \frac{S_0}{(e^{S_1 \cdot (T_e + T_p - S_2)} + 1) \cdot (e^{S_5 \cdot (t - S_6)} + 1) \cdot (e^{S_3 \cdot (-t - S_4)} + 1)} \quad \text{SLBP}$$
$$V_5(t) = \frac{A_0}{(e^{A_1 \cdot (t - A_2)} + 1) \cdot (e^{A_3 \cdot (-t - A_4)} + 1)} \quad \text{DNA synthesis}$$

Figure A: Full set of equations to describe the extended histone feedback loop model. The equations and notations coincide with the regulatory scheme shown in Figure 5 of the main text.

$$v_{1e2b} = S(t) \cdot V_{max,e2b} \cdot \frac{D^n}{K_Y^n + D^n} \qquad \text{endog. H2B RNA synthesis}$$

$$v_{1p2b} = V_{max,p2b} \qquad \text{exog. H2B RNA synthesis}$$

$$v_{1e3} = S(t) \cdot V_{max,e3} \cdot \frac{D^n}{K_Y^n + D^n} \qquad \text{endog. H3 RNA synthesis}$$

$$v_{2e2b} = \left(\gamma_{base} + \gamma_{ind} \cdot \frac{1}{1 + \left(\frac{D}{I_Y}\right)^m} \right) \cdot R_{e2b} \qquad \text{endog. H2B RNA degradation}$$

$$v_{2p2b} = \gamma_{base} \cdot R_{p2b} \qquad \text{exog. H2B RNA degradation}$$

$$v_{2e3} = \left(\gamma_{base} + \gamma_{ind} \cdot \frac{1}{1 + \left(\frac{D}{I_Y}\right)^m} \right) \cdot R_{e3} \qquad \text{endog. H3 RNA degradation}$$

Figure B: Alternative equations for the extended DNA coupled model. Only the fluxes of the endogenous RNA synthesis and degradation are affected by the different regulations. The other equations are the same as given in Figure A for the histone feedback loop model.

Reference List

1. Sittman DB, Graves RA, Marzluff WF: **Histone mRNA concentrations are regulated at the level of transcription and mRNA degradation.** *Proc Natl Acad Sci USA* 1983, **80:** 1849-1853.

2. Heintz N, Sive HL, Roeder RG: **Regulation of human histone gene expression: kinetics of accumulation and changes in the rate of synthesis and in the half-lives of individual histone mRNAs during the HeLa cell cycle.** *Mol Cell Biol* 1983, **3:** 539-550.

3. Hameister H: *Mathematical models for DNA replication machinery.* University of Aberdeen; 2012. available http://ethos.bl.uk/OrderDetails.do?uin=uk.bl.ethos.558612

4. Clark D: *Molecular Biology*. Elsevier; 2005.

5. Strachan T, Read A: *Human Molecular Genetics*. Garland Science; 2003.

6. DeLisle AJ, Graves RA, Marzluff WF, Johnson LF: **Regulation of histone mRNA production and stability in serum-stimulated mouse 3T6 fibroblasts.** *Mol Cell Biol* 1983, **3:** 1920-1929.

7. Harris ME, Böhni R, Schneiderman MH, Ramamurthy L, Schümperli D, Marzluff WF: **Regulation of histone mRNA in the unperturbed cell cycle: evidence suggesting control at two posttranscriptional steps.** *Mol Cell Biol* 1991, **11:** 2416-2424.

8. Morris TD, Weber LA, Hickey E, Stein GS, Stein JL: **Changes in the stability of a human H3 histone mRNA during the HeLa cell cycle.** *Mol Cell Biol* 1991, **11:** 544-553.
