# Supplementary material for: Modelling Robust Feedback Control Mechanisms That Ensure Reliable Coordination of Histone Gene Expression with DNA Replication
Source: PLoS One. 2016 Oct 31;11(10):e0165848. doi: 10.1371/journal.pone.0165848 (PMC5087906; doi:10.1371/journal.pone.0165848)
Supplement: S2 File — (PDF) [file pone.0165848.s003.pdf]

S2 File: The post processing of the flow cytometry data from synchronised cell cultures to derive the V_5 time course for the mathematical model

Processing of raw data obtained by cell cycle analysis using flow cytometry

We explain here the post processing, data analysis and fitting of the cell cycle analysis performed with human U2OS cells released from double thymidine block. DNA replication and cell cycle progression were analysed by BD Biosciences LSRII flow cytometry at the University of Aberdeen Iain Fraser Cytometry Centre, using the BD Pharmingen APC BrdU Flow Kit for pulse-labelling with BrdU and staining with 7-aminoactinomycin D (7-AAD) to measure DNA synthesis and DNA content, respectively. The result of this analysis was stored in the on-site data format *.fcs*. In a first step we converted the raw data with the program *FlowJo* into *.csv* format. Upon import into *Excel* header lines were removed, leaving only the plain data in a two column structure, the 7-AAD signal in the first and the BrdU signal in the second column. The data was then saved as a *Windows* formatted text. This series of steps was performed with data generated at each time point. As a next step we imported the data into *Matlab*™ and calculated a density plot with the built-in function *hist3*. Extreme high or low data points were excluded by removing points that were outside the following parameters: $15000 \leq 7\text{-}AAD \leq 140000$ and $30 \leq BrdU \leq 20000$. This selection excluded ≤5% of data points from the ensemble of 100000 data points at each time point. The density plots are shown in Fig. A.

G_1 phase cells as reference for a linear rescaling

The cells in G_1 phase, located in the lower left corner of each plot, were used as a reference for a quantitative comparison. The density plots showing the raw data (Fig. A) reveal that the position of the cells in G_1 varied from one time point to the next. As a consequence, the raw data were not quantitatively comparable in this form. As this analysis is a complex procedure with several critical steps ranging from the synchronisation procedure with two cell cycle blocks, the pulse labelling with BrdU, the staining with 7-AAD to the analysis by flow cytometry, subtle differences between samples can be expected and will affect the intensity of the signals measured by flow cytometry. We assume that BrdU and 7-AAD signals are affected in a linear manner, and therefore a single standardisation factor for

each time point will be able to compensate these differences. To determine this standardisation factor we selected at each time point the G_1 phase cell population and identified the maximum 7-AAD and BrdU signal in these cells. We choose the borders for the G_1 phase cell population as listed in Table A and identified the peak 7-AAD and BrdU signals by plotting histograms of the 7-AAD and BrdU signals for each time point (Fig. B, Table B). We arbitrarily choose the G_1 cell population of the 4h time point as reference and calculated factors to match the 7-AAD and BrdU peaks of all G_1 phase cell populations to the *4h* reference, by dividing the *4h* peak value by these respective peak values. These standardisation factors are listed in Table B.

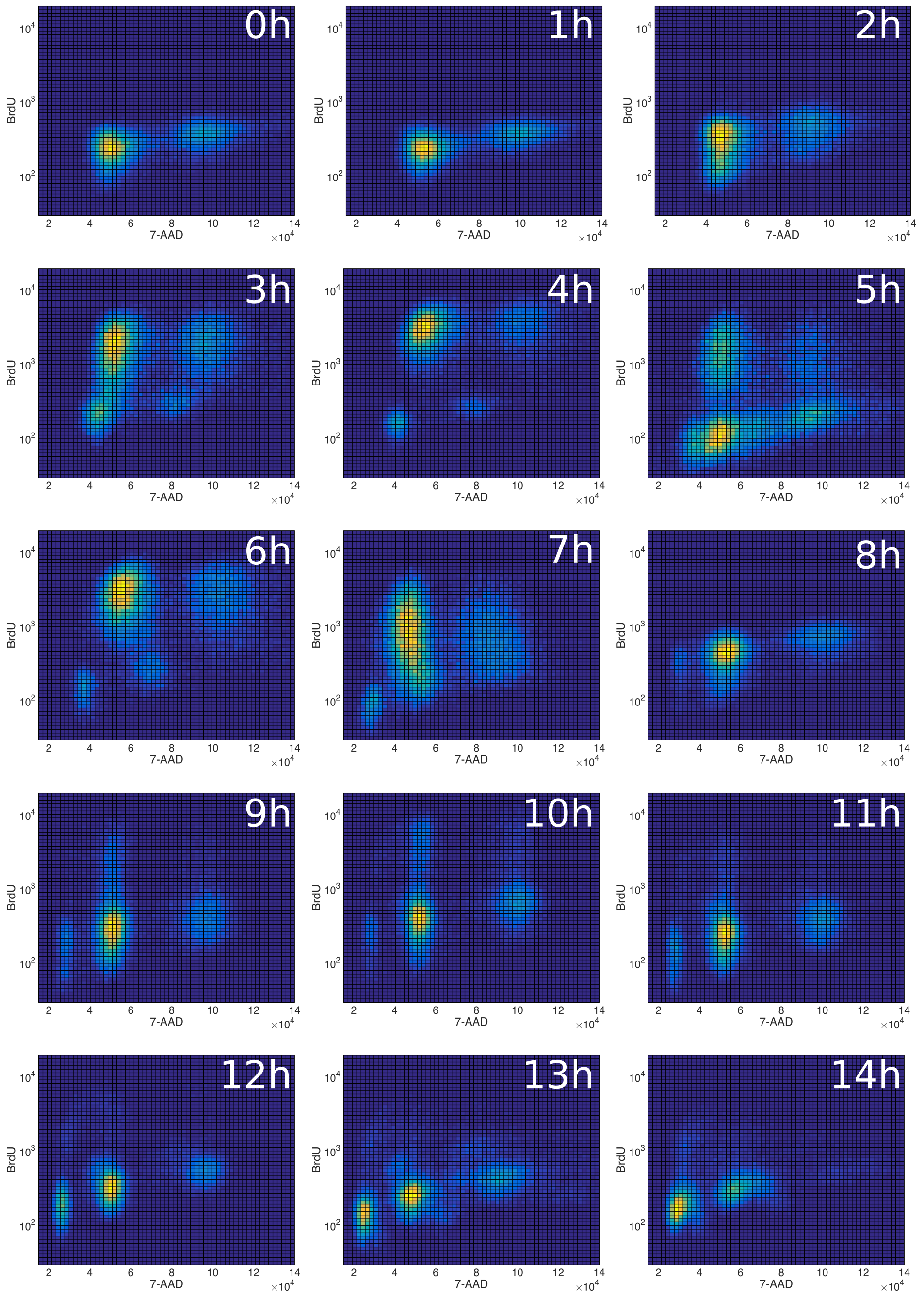

Figure A: Analysis of cell cycle progression. Density plots of the raw data obtained by flow cytometry. The y-axis shows BrdU labelling in logarithmic scale ranging from 30 to 20000 and the x-axis shows 7-AAD labelling in linear scale from 15000 to 140000. The cell number in each box is colour coded ranging from dark blue for low cell numbers to light yellow for high cell numbers. The time points were sampled every hour beginning at time 0 h on the top left panel with increasing time from left to right and top to bottom up to 14 h on the bottom right panel. All panels are scaled in the same range.

At this stage, cells with a 7-AAD value >100000, which represent cell doublets, were excluded from the analysis. The standardised density plots for all time points are shown in Fig. C and illustrate the doubling of DNA content (7-AAD) during S phase from 4 to 8 on the x axis (corresponding to doubling of DNA from 2n to 4n) and the DNA replication activity (BrdU) during this experiment.

time point	7-AAD		BrdU		# cells in G_1
	min	max	min	max	
0	30000	70000	50	1000	60294
1	35000	70000	50	1000	59714
2	30000	66000	50	1000	63865
3	30000	59000	50	1000	11576
4	30000	50000	50	300	7518
5	28000	78000	30	290	37999
6	28000	45000	50	300	4854
7	20000	35000	30	220	5106
8	23000	35000	40	1500	5613
9	20000	35000	30	800	8004
10	20000	34000	30	800	5224
11	20000	34000	30	600	9545
12	20000	31000	50	500	16859
13	18000	31000	40	400	18633
14	22000	42000	50	600	40070

Table A: 7-AAD and BrdU limits of G_1 phase cell populations shown in Fig. A.

As described above the G_1 phase cell population was used as reference. In Figure C, the 7-AAD and BrdU signals of S phase and G_2 phase cell populations have become consistent between time points. This is a consequence of the application of a standardisation factor determined using G_1 phase cells and was not part of the target fit function. This strengthens the conjecture that the major uncontrolled processes affect the fluorescence signal like a simple linear gain factor.

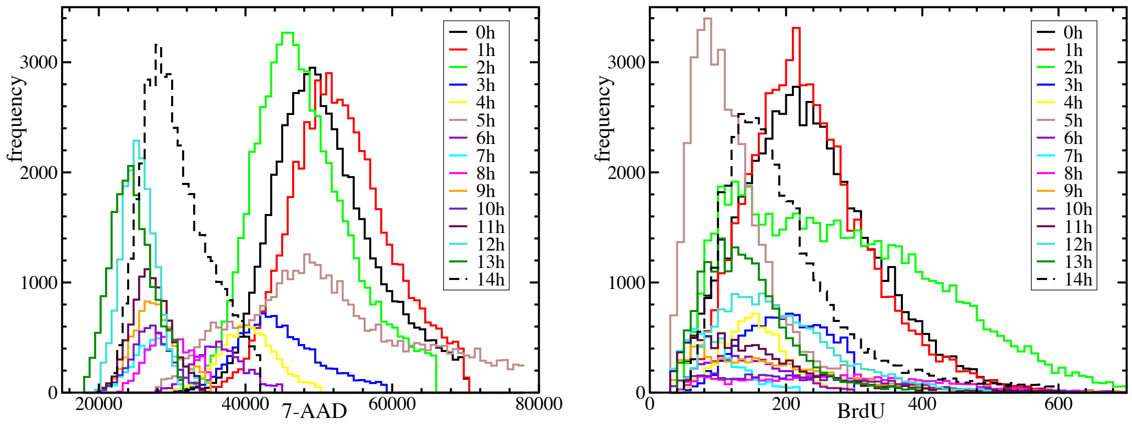

Figure B: Histogram of the 7-AAD (left) and the BrdU (right) signal distribution in *G_1* phase cell populations selected according to the limits listed in Table A.

We tested the quantitative quality of the results also by merging all data points in one file and plotting a single density plot for all cells at all time points (Fig. D). In contrast to the combination of the raw data (left panel) the standardisation (right panel) leads to the typical horseshoe pattern characteristic for an unsynchronised cell population with cells at all stages of the cell cycle. This confirms that our mathematical re-scaling (standardisation) of the data allows quantitative comparisons between different time points.

A second test in Figure E shows histograms of the 7-AAD raw data (left panel) and after standardisation (right panel) for each time point. The histograms were generated for the same bin width of 750 units of the 7-AAD signal and stacked vertically from bottom to top to represent time progression. We assigned the first peak to cells in G_1 with 2n DNA content and standardised the entire population with respect to these cells. After the standardisation procedure the histograms express the typical and expected change of DNA content through S phase and then mitosis, with doubling of the DNA content to 4n followed by re-appearance of cells with 2n DNA content after mitosis. The time progressions into S phase and further into G_2 with the corresponding changes in DNA content result from this and were not part of the standardisation aim.

Determination of median 7-AAD and BrdU values at each time point

The density plots produced from the standardisation data (Fig. C) show a significant diversity amongst the cells for each sample analysed. Focusing on a specific group of cells such as for example cells in

S phase would involve arbitrariness in setting the limits and diminish the value of the measurement for the mathematical model, especially for the transition from G_1 into S phase and from S into G_2 phase. Hence we took into account all cells as shown in Fig. C and calculated the median values of the 7-AAD and the BrdU signals. We chose to use the median value to reflect the behaviour of the whole cell population including cells entering S phase early or late.

**time**	**7AAD**		**BrdU**	
**point**	**peak**	**factor**	**peak**	**factor**
0	48100	0.84	218	0.67
1	52000	0.78	218	0.67
2	45700	0.88	130	1.12
3	43300	0.93	206	0.71
4	40300	1	146	1
5	36300	1.11	90	1.62
6	36300	1.11	125	1.17
7	28000	1.44	73	2
8	29400	1.37	73	2
9	27100	1.49	73	2
10	27100	1.49	123	1.19
11	26800	1.50	100	1.46
12	25300	1.59	150	0.97
13	24200	1.67	112	1.30
14	28350	1.42	147	0.99

Table B: G_1 cell population 7-AAD and BrdU peak signals and the standardisation factors for direct comparison with the 4h time point data.

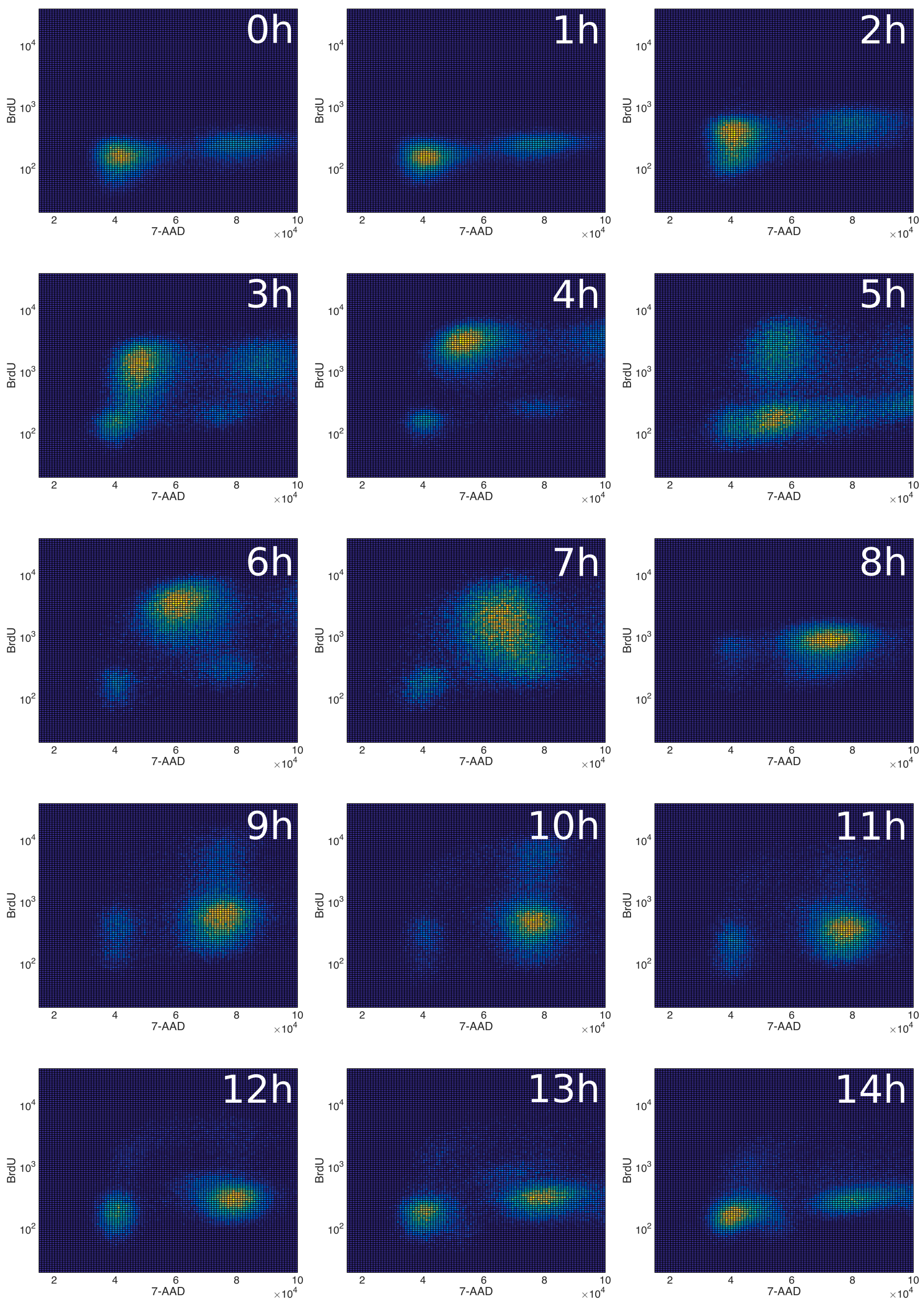

Figure C: Standardised data from flow cytometry after linear rescaling with the G_1 phase as reference (compare with Fig. A). All panels are scaled in the same range.

The results are shown in Figures F and G and in Table C, where median values calculated from the raw data are also included to illustrate the effect of the data rescaling applied.

Fig. F shows the progression of the 7-AAD (left panel) and BrdU signal (right panel) during the

experiment. The median calculated from the 7-AAD raw data (blue) does not reflect the increase in DNA content known to happen during S phase. In contrast, after processing, the data clearly shows the continuous accumulation of DNA during S phase, reaching a plateau in G_2 phase cells of twice the content of G_1 phase cells when DNA replication is completed. The decrease in DNA content at the 14 h time point reflects the progression of cells into M phase.

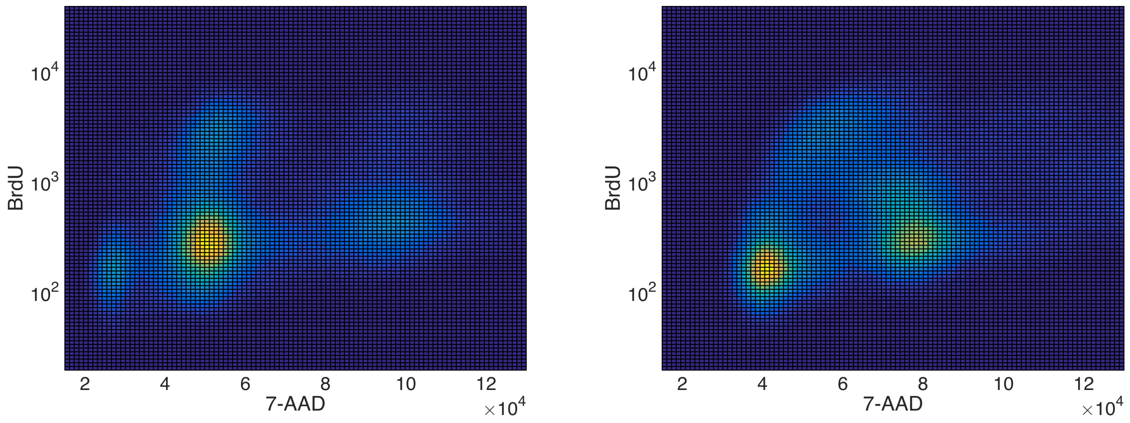

Figure D: Comparison of raw and standardised flow cytometry data. The left-hand side density plot displays the combined unmodified raw data from all time points. The right-hand side density plot displays the combined standardised data from all time points. 7-AAD staining is plotted on the x-axis, the BrdU labelling is shown on the logarithmically scaled y-axis. Cell density is colour coded ranging from dark blue for low cell density to light yellow for high cell density.

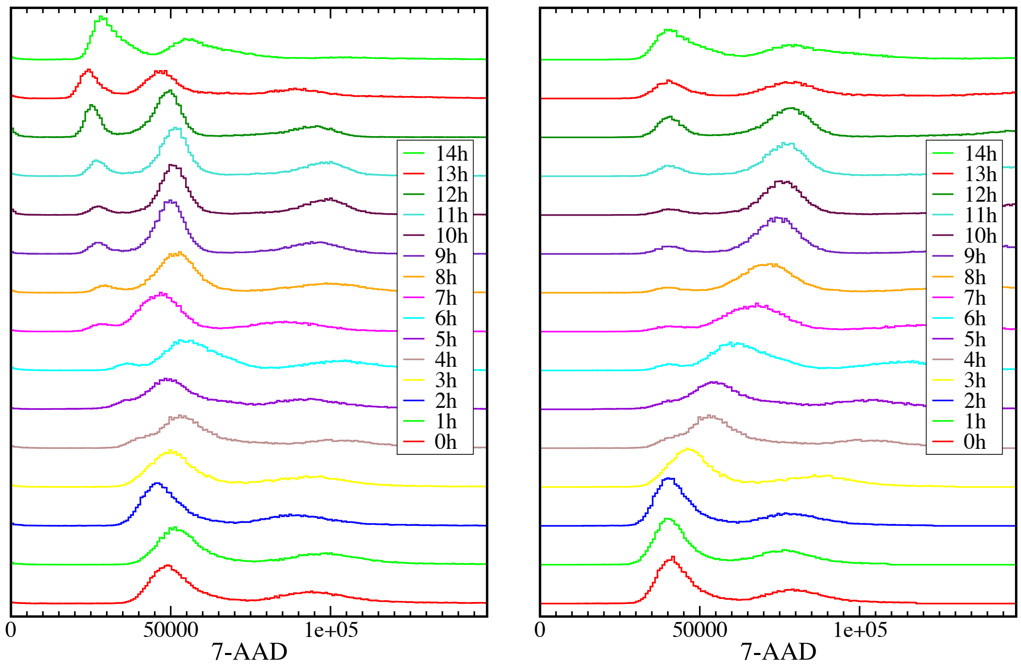

Figure E: 7-AAD histogram of the raw data (left) and the standardised signal (right). The results for different time points are stacked with increasing time from bottom to top.

Figure F shows that the BrdU signal is more consistent between time points and is not as strongly affected by the data processing as the 7-AAD signal. DNA replication, as measured by BrdU incorporation, peaks between 3 h and 8 h. However at the 5 h time point the BrdU signal is low and similar to G_1 phase cells. This is also reflected in the density plot in Fig. C with a significant population of cells with DNA content between the G_1 and the G_2 phase cells but with a low BrdU signal. We have observed this reduction in BrdU incorporation during S phase in two independent experiments but are not in a position to explain this effect. As this was not observed in earlier cell cycle analyses by others (see e.g. analysis of DNA synthesis during S phase using radiolabelled nucleotides [1,2]) we have omitted this data point from the subsequent analysis and data fitting.

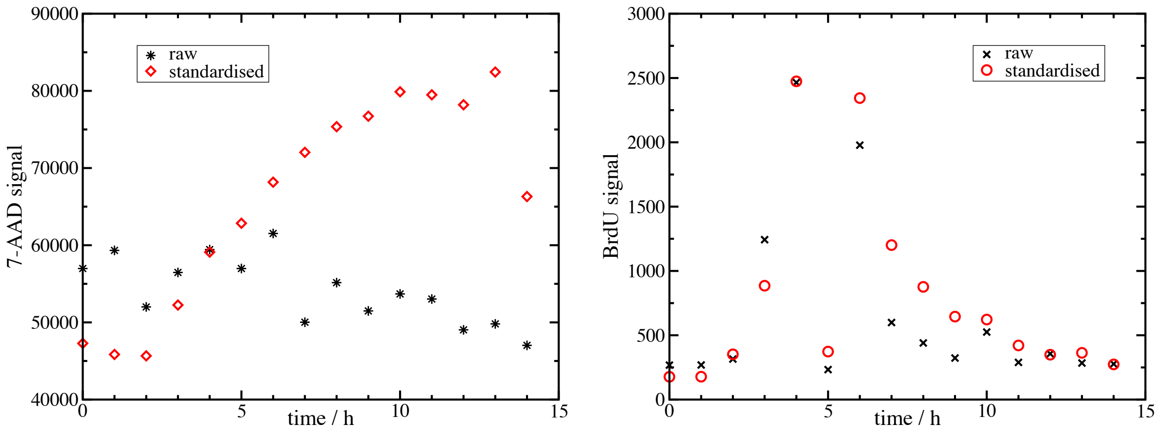

Figure F: Time course of DNA content and DNA synthesis. Median values of the 7-AAD signal (left panel) and the BrdU signal (right panel) derived from the analysis by flow cytometry were plotted against time. Both panels include the raw data (black symbols) and the standardised data (red symbols).

time point	# cells used	Median	
		7-AAD	BrdU
0	93713	46758	175
1	95171	45618	178
2	95214	45279	346
3	91729	51121	867
4	81585	56177	2408
5	76267	57804	312
6	71767	62804	2258
7	70841	66657	1174
8	68984	70340	770
9	72052	73010	575
10	63409	74810	499
11	67589	74873	350
12	72933	73717	291
13	60859	70699	280
14	82753	53147	248

Table C: Median values of the 7-AAD and BrdU signals after rescaling and exclusion of doublets. The second column indicates the numbers of cells (out of 100000) fitting to the selection criteria. The median values were based on these populations.

Standardising the BrdU signal reflecting DNA replication with respect to DNA content

The BrdU signal directly reflects DNA synthesis. It serves as an essential experimental input for the mathematical model and is linked to the V_5 flux. Therefore the BrdU time course was approximated using an arbitrary fit function. This was complicated by the fact that median BrdU values at the beginning and at the end of the experiment were not similar (compare BrdU values at 0h and 1h with values at 12h and 13h). This is also reflected in the density plots (Figs. C and D right panel) where the BrdU signal is higher in G_2 phase cells than in G_1 phase cells, possibly reflecting a higher rate of background (repair) DNA synthesis in cells with 4n DNA content, or unspecific binding of the antibodies. In Figure G the BrdU signal is plotted as function of the 7-AAD signal for each time point. The lines connect points with progressing time from the bottom left corner for G_1 cells at early time points, to the S phase in the centre and the G_2 phase at the end of time course shown at the bottom right corner. As before, the raw data did not reflect the changes of BrdU und 7-AAD signals expected during this time course (black line and symbols in Fig G, left) but the data processing (standardisation and doublet exclusion) resulted in a typical horseshoe like curve. The low BrdU signal in the centre represents the 5 h time point discussed before. The 7-AAD -dependent BrdU offset can also be seen in the 7-AAD-BrdU plot (Fig. G) and might be caused for example by genome size dependent continuous basal DNA repair processes. To correct for this DNA-content dependent effect on BrdU incoorporation we removed this offset from the data before fitting the V_5 curve. We assumed that the BrdU offset depends linearly on the 7-AAD signal and fitted a linear curve trough the data points at early time belonging to G_1 (first two time points at 0h and 1h), to G_2 and to M phase (last three time points at 12h, 13h, and 14h). The resulting linear fit function $[BrdU] = -68.035 + 0.0052494 \times [7\text{-}AAD]$ is shown in green in Figure G (right panel). Subtracting the linear offset function at each time point leads to the offset-free data shown in blue, which was used for the V_5 curve fit. Please note that the 5h time point was omitted from the curve fitting.

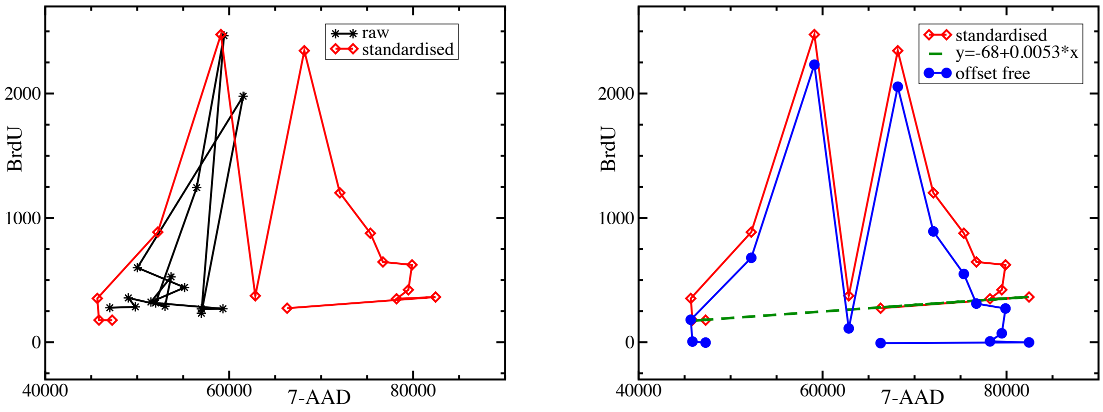

Figure G. Two-dimensional representation of median BrdU and 7-AAD signals. Left: calculated from the raw (black) and from the standardised flow cytometry data (red). Right: Two-dimensional representation showing median BrdU and 7-AAD signals calculated using the standardised data without (red) and after offset subtraction (blue). The linear curve used to calculate the offset is shown in green colour.

Fitting of the $V_5(t)$ function

The synthesis of new DNA is a major input into our mathematical model. To enter the experimental results into the mathematical model we need a continuous mathematical formula fitting the BrdU time course. The proposed mathematical model focuses on the regulation of the histone protein synthesis and do not take into account the precise mechanisms of DNA replication. We fit the BrdU time course with an arbitrary function. The fit function must approximate the initial increase in DNA replication and the subsequent decline. We tested Gaussian, Lorenzian, Morris-Lecar (1/*cosh(x)*) and the plateau e-function to fit the experimental data. The plateau e-function produced the best approximation to both data sets available (sampling every hour in experiment 1, discussed in detail herein and data from a second experiment with 45 min intervals between sampling, not shown in great detail) with the function:

$$y = f(x) = \frac{A0}{\left(e^{A1(x-A2)}+1\right)\left(e^{A3(-x-A4)}+1\right)} ,$$

with time as the independent variable x and BrdU as the dependent variable y. The plateau e- function without offset has five free fit parameters; A0, A1, A2, A3 and A4. We deduced a non-linear curve fitting with the free data analysis and plotting program *xmgrace*. The fit results are A0=2300 min^{-1}, A1=0.0216528 min^{-1}, A2 = 420.199 min, A3 = 0.0753725 min^{-1} and A4 = −191.239 min with a correlation

coefficient 0.985586 showing a good match between fit curve and data points (Fig. H, left panel). We assume that the BrdU time course directly reflects DNA replication. The flux V_5 in the mathematical model defines the number of histone proteins necessary to pack the newly build DNA. The flux V_5 is directly linked to the newly synthesised DNA and so qualitatively comparable with the BrdU signal from the flow cytometry. We estimate that 3.4×10^7 histone proteins of each core histone type must be synthesised during S phase in human cells to ensure correct packaging of the newly synthesised DNA (see Table B in supplementary S1 File). Consequently the integral of the flux V_5 over the entire S phase must end up with ~3.4×10^7 histone proteins. This leads to the new rescaled parameter $A0_{new}$ = 148258 min^{-1}. We execute the numerical simulations in seconds as time unit and all parameters from the literature and our experiments must be expressed accordingly. This leads to the following experimentally derived control parameters (rounded to three significant figures); $A0_{new}$ = 2470 s^{-1}, A1 = 0.000361 s^{-1}, A2 = 25200 s and A3 = 0.00126 s^{-1} and A4=-11500 s for the function

$$V_5(t) = \frac{A0_{new}}{\left(e^{A1(t-A2)}+1\right)\left(e^{A3(-t-A4)}+1\right)} \quad ,$$

giving the production of new DNA as binding sites for histones as a function of time *t* for the mathematical model.

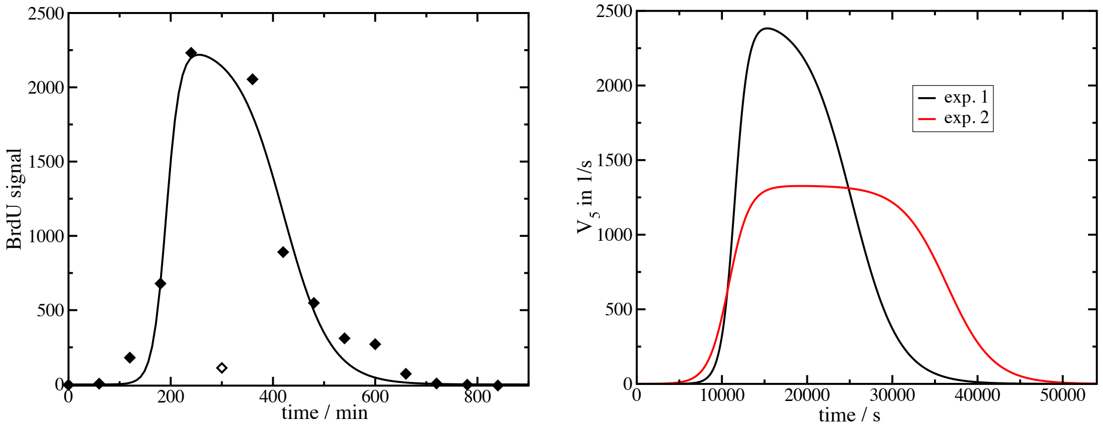

Figure H: Fitting of V_5(t) function. Left: Fitting of the BrdU time course with the plateau e-function. Shown are BrdU signal (diamonds) at the indicated time points (min). The result of the curve fitting is shown as a solid line. The 5h value (open diamond) was omitted from curve fitting. Right: Time evaluation of the DNA synthesis V_5 derived from the BrdU flow cytometry data from experiment 1 (black), described in details in this supplementary, and experiment 2 with 45 min sampling (red).

Fig. H (right panel) shows the resulting V_5(t) curve appropriately scaled as input for the mathematical model. The black curve has been derived from the experiment analysed here (exp. 1) and the red curve

is the product of the equivalent treatment applied to our second experiment (exp. 2) where cells were analysed every 45 min. Both curves are quantitatively different and illustrate the diversity in the cell cycle and DNA replication dynamics between experiments. These different experiments represent a good test for the ability of the mathematical model to deal with different conditions without changing other model parameters. They allow us to demonstrate the meaningfulness and flexibility of the model we propose in the main manuscript.

The 7-AAD signal, as the second fluorescence signal, reflects the total DNA content in the cells. The total DNA content over time was not used for the parameterisation of the mathematical model but can be used for its validation. We will use the mathematical model to predict the total new DNA over time and compare the model predictions with the experimental flow cytometry measurement.

Reference List

1. Plumb M, Stein J, Stein G: **Coordinate regulation of multiple histone mRNAs during the cell cycle in HeLa cells.** *Nucl Acids Res* 1983, **11:** 2391-2410.

2. Plumb M, Marashi F, Green L, Zimmerman A, Zimmerman S, Stein J *et al.*: **Cell cycle regulation of human histone H1 mRNA.** *Proc Natl Acad Sci USA* 1984, **81:** 434-438.
