# Supplementary material for: Modelling Robust Feedback Control Mechanisms That Ensure Reliable Coordination of Histone Gene Expression with DNA Replication
Source: PLoS One. 2016 Oct 31;11(10):e0165848. doi: 10.1371/journal.pone.0165848 (PMC5087906; doi:10.1371/journal.pone.0165848)
Supplement: S3 File — (PDF) [file pone.0165848.s004.pdf]

S3 File: The data analysis of the Northern blots and the derivation of the degradation constants for the mathematical model

Analysis of Histone RNA half-life.

Here we analyse the effect of inhibition of DNA replication on histone H2B RNA levels in U2OS cells. To differentiate between the effects of the DNA replication inhibitor hydroxyurea (HU) on transcription and on RNA stability we have included a pre-treatment with the transcription inhibitor actinomycin D (ActD). Cells were synchronised and then treated with/without ActD, and 15 min later with or without HU. We are aware that ActD inhibits RNA synthesis by binding DNA, and that this can also affect DNA replication [1,2]. To minimise any impact, ActD was titrated and used at a concentration of 4 µM. This ensured robust inhibition of histone RNA synthesis, but after extended incubation (90 min) also affected DNA replication in U2OS cells (data not shown). However, our analysis (see Figure A) was able to differentiate between effects of ActD and HU on histone RNA levels. U2OS cells were lysed and RNA was isolated at the indicated times and analysed by Northern blotting. Histone H2B RNA levels and, as reference, GAPDH RNA levels were measured (see Material and Methods for details). For quantitation as shown for example in Figure A Panel B, the levels of H2B RNA were standardised using GAPDH RNA as reference.

We assume that H2B RNA decay follows single exponential decay. Single exponential decay is displayed as a linear decay curve in logarithmic scaling and the slope of a linear fit gives the decay constant. Figure A shows the natural logarithm of the standardised H2B RNA levels versus time (Figure A Panel B, left hand panel). The free software *xmgrace* was used for the standard linear fit according to the function $y = ax + b$, with the fit parameter slope a and offset b, the independent variable x as time in min and the dependent variable y as the logarithm of the H2B RNA signal. The fitted curves are shown as solid lines in Figure A Panel B (right), and Table A summarises the fit results. In the control experiment without any drug treatment, H2B RNA levels (blue circles) were largely similar throughout the experiment except for the 30 min time point, which was omitted from the analysis. In cells treated with ActD only (red diamonds), H2B RNA levels decreased from the 10 min time point onward, and only data points between 10 min and 60 min were used for curve fitting. Data from this time course was used for an approximation of histone H2B RNA stability under undisturbed conditions. Figure A Panel B shows a clear exponential decay in the linear scaled graph and linear decay in the logarithmic scaled graph. In

cells treated with HU only, H2B RNA (black triangles) decreased from the 15 min time point onwards and time points between 10 min and 60 min were used for curve fitting. In cells treated with both ActD and HU, reduction of H2B RNA levels (green squares) followed a biphasic pattern. Phase 1, early time points between 10 min and 30 min, was fitted separately from phase 2, which spans time points between 30 min and 60 min. The 10 min to 30 min phase 1 section matches very well with the curve derived from cells treated with ActD only while phase 2 reflects an increased reduction of H2B RNA levels similar to the one observed with HU only between 30 min and 60 min.

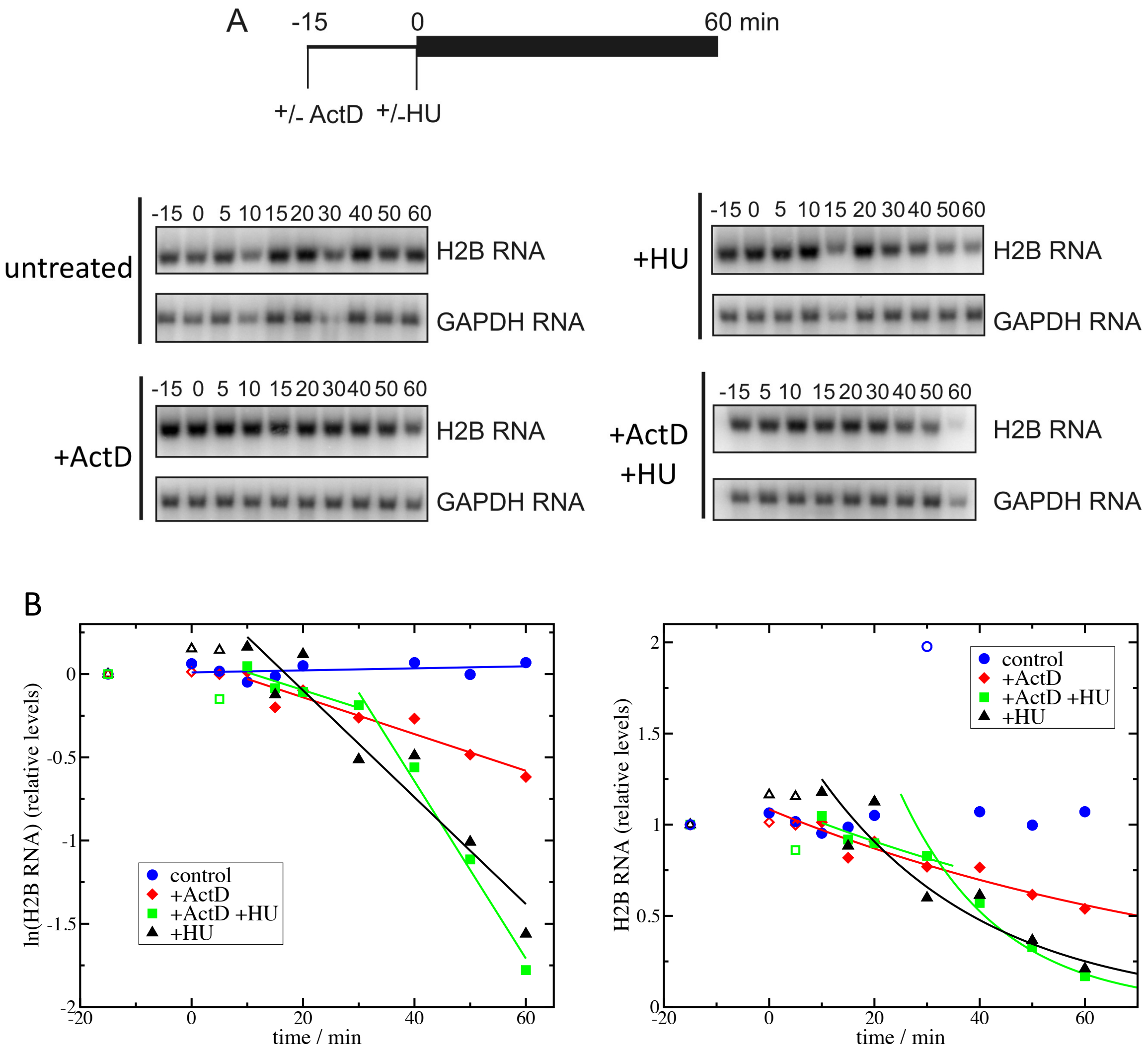

Figure A: Analysis of H2B RNA decay. Panel A. Analysis of histone H2B RNA levels by Northern blotting. The diagram illustrates the experimental design. ActD (4 µM) and/or HU (5 mM) were added at the indicated time points to synchronised U2OS cells released for 4 h into S phase. Samples were taken at the indicated time points, with the time of HU addition arbitrarily set as 0 min. Histone H2B and GAPDH RNA levels were analysed by Northern blotting. H2B RNA levels were standardised using GAPDH RNA levels and the levels at the -15 min time point were defined as 1. Note that the 0 min time point from cells treated with ActD and HU is missing. As histone H2B RNA levels decrease later, this does not affect our analysis. Panel B. The data obtained from (Panel A) were plotted in graphs with linear Y-axis (left) and Y-axis with a logarithmic scale (right). The symbols show the results from the Northern Blots and the lines the curves fitted assuming single exponential decay. Only data points with filled symbols were used for the curve fitting.

experiment	slope *a* in min $^{-1}$	Offset *b*	Number of data points used for the fit	minimal time point used	maximal time point used
control	0.00061 ±0.00077	0.010 ±0.025	8	0 min	60 min
+ ActD	-0.0110 ±0.0016	0.081 ±0.059	7	10 min	60 min
+ HU	-0.0321 ±0.0043	0.544 ±0.156	7	10 min	60 min
+ ActD + HU (phase 1)	-0.0107 ±0.0028	0.117 ±0.057	4	10 min	30 min
+ ActD +HU (phase 2)	-0.0533 ±0.0046	1.488 ±0.215	4	30 min	60 min

Table A: Summary of the linear curve fitting results using data from the experiment described in Figure A.

Parameterisation of the mathematical model.

The mathematical model includes a basal RNA degradation rate γ_{base} at all times, and an induced degradation rate activated gradually by an excess of free histones (histone feedback loop model) or inhibited by free DNA (DNA coupled model) and described by Hill type kinetics. The Hill functions varies between zero and one depending on the free histone protein concentration (or DNA) and the induced degradation factor γ_{ind} defines the maximal induced degradation rate. H2B RNA levels are determined by synthesis, the basal RNA degradation rate and the free histone protein-dependent (or DNA dependent) induced RNA degradation rate. The H2B RNA degradation in ActD treated cells in Figure A reflects the basal degradation. The slopes *a* derived from cells treated with ActD only and the initial rate derived

from cells treated with ActD and HU are very similar, with 0.011026 min^{-1} and 0.0106551 min^{-1}, respectively (Table A, +ActD +HU, phase 1). We used the mean value of these two, rounded to three significant numbers (0.0108 min^{-1}) as γ_{base} in the mathematical model. As the mathematical model is scaled in seconds as the general time unit, the basal degradation rate γ_{base} is 0.00018s^{-1} (Table A in supplementary S1 File,). HU inhibits DNA synthesis, and, by interrupting the incorporation of histone proteins into chromatin, leads to an excess of free histone proteins and the activation of the induced histone RNA degradation process. In our model, the effect of treatment with HU on the histone RNA degradation rate is integrated by increasing the Hill functions controlling the induced degradation gradually to one. In this way the histone RNA decay in the model is determined by the induced degradation rate γ_{ind} and the basal degradation rate γ_{base}.

In cells treated with ActD and HU, the effect of HU becomes apparent at later times in the time course (Figure A). This is confirmed by the larger slope a in the later part of this experiment (Table A, +ActD+HU, phase 2), which is similar to slope a determined in cells treated with HU only (0.0533 min^{-1} and 0.0321 min^{-1}, respectively). Rounding a from the combined experiment and subtracting the basal degradation derived from the ActD treatment gives an induced degradation rate $\gamma_{ind} \approx 0.04$ min^{-1} or $\gamma_{ind} \approx 0.00067$ s^{-1} if scaled in seconds as appropriate for the mathematical model. The degradation constants correspond to a histone RNA half-life of 64 min and 17.3 min for the basal degradation rate γ_{base} and the induced degradation rate γ_{ind}, respectively. The combination of ActD and HU treatment leads to a half-life of 63 min in phase 1 and 13 min in phase 2.

Histone RNA half-life in S phase has been previously measured in CHO, HeLa and mouse 3T6 cells and found to vary between 45 min and 4h -5h, depending on the cell type [3-5]. Our estimate for the histone mRNA half-life in S phase U2OS cells of 64 min lies between the values reported for CHO cells (45 min, [3]) and HeLa cells (110 min, [4]). Inhibition of S phase by HU and other compounds that inhibit DNA replication is known to shorten histone RNA half-life. In mouse myeloma and HeLa cells the histone RNA half-life under such condition is between 10 min and 15 min [6,7], which is similar to the half-life of 17.3 min observed in U2OS cells when DNA replication is inhibited in S phase.

Model predictions of alteration of histone RNA levels in response to inhibition of transcription and DNA replication.

The basic mathematical model with regulation by the free histone protein pool (see main text, Figure 1)

was constructed to describe and investigate the normal changes occurring when cells transit from G_1 phase through S phase into G_2 phase. This mathematical model is able to predict the effects of treatment with the DNA replication inhibitor HU and the transcription inhibitor ActD (Figure B). For predictions of the H2B RNA decay we set the regulators V_5 and S in the mathematical model constant to 1200 and 1 respectively. We discard a short transient to allow the mathematical model to reach a steady state independent of the initial conditions. From the steady state we set the RNA synthesis flux $v_1 = 0$ and the DNA replication rate $V_5 = 0$ to mimic treatment with ActD and HU, respectively. To reproduce the experiment in Figure A, the treatment with ActD was applied prior to the HU treatment in the mathematical model. To compare predictions and experiment, we standardised the RNA levels predicted by the mathematical model as was done for the analysis by Northern blotting. The mathematical model (Figure B) is in good agreement with the data from the experiment described in Figure A.

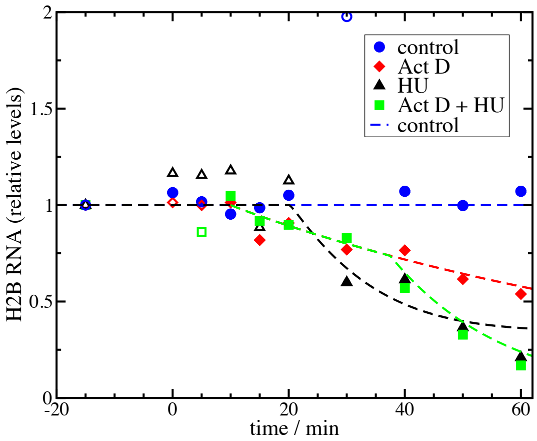

Figure B. Model prediction of the effect treatment with ActD and HU on H2B RNA levels. Shown are histone RNA levels from the experiment described in Figure A (symbols) and predictions by the mathematical model (dashed lines).

Reference List

1. Guy AL, Taylor JH: **Actinomycin D inhibits initiation of DNA replication in mammalian cells.** *Proc Natl Acad Sci U S A* 1978, **75:** 6088-6092.

2. Robinson H, Gao YG, Yang Xl, Sanishvili R, Joachimiak A, Wang AHJ: **Crystallographic Analysis of a Novel Complex of Actinomycin D Bound to the DNA Decamer CGATCGATCG.** *Biochemistry* 2001, **40:** 5587-5592.

3. Harris ME, Böhni R, Schneiderman MH, Ramamurthy L, Schümperli D, Marzluff WF: **Regulation of histone mRNA in the unperturbed cell cycle: evidence suggesting control at two posttranscriptional steps.** *Mol Cell Biol* 1991, **11:** 2416-2424.

4. Morris TD, Weber LA, Hickey E, Stein GS, Stein JL: **Changes in the stability of a human H3 histone mRNA during the HeLa cell cycle.** *Mol Cell Biol* 1991, **11:** 544-553.

5. DeLisle AJ, Graves RA, Marzluff WF, Johnson LF: **Regulation of histone mRNA production and stability in serum-stimulated mouse 3T6 fibroblasts.** *Mol Cell Biol* 1983, **3:** 1920-1929.

6. Sittman DB, Graves RA, Marzluff WF: **Histone mRNA concentrations are regulated at the level of transcription and mRNA degradation.** *Proc Natl Acad Sci USA* 1983, **80:** 1849-1853.

7. Baumbach LL, Marashi F, Plumb M, Stein G, Stein J: **Inhibition of DNA replication coordinately reduces cellular levels of core and H1 histone mRNAs: requirement for protein synthesis.** *Biochemistry* 1984, **23:** 1618-1625.
